# TMV-Cg Coat Protein stabilizes DELLA proteins and in turn negatively modulates salicylic acid-mediated defense pathway during *Arabidopsis thaliana* viral infection

**DOI:** 10.1186/s12870-014-0210-x

**Published:** 2014-08-03

**Authors:** Maria Cecilia Rodriguez, Gabriela Conti, Diego Zavallo, Carlos Augusto Manacorda, Sebastian Asurmendi

**Affiliations:** 1 Instituto de Biotecnología, CICVyA-INTA, Hurlingham, 1686, Buenos Aires, Argentina; 2 Consejo Nacional de Investigaciones Científicas y Técnicas (CONICET), Hurlingham, Buenos Aires, Argentina

**Keywords:** TMV-Cg, Coat protein, DELLA proteins, SA signaling, Defense response

## Abstract

**Background:**

Plant viral infections disturb defense regulatory networks during tissue invasion. Emerging evidence demonstrates that a significant proportion of these alterations are mediated by hormone imbalances. Although the DELLA proteins have been reported to be central players in hormone cross-talk, their role in the modulation of hormone signaling during virus infections remains unknown.

**Results:**

This work revealed that TMV-Cg coat protein (CgCP) suppresses the salicylic acid (SA) signaling pathway without altering defense hormone SA or jasmonic acid (JA) levels in *Arabidopsis thaliana*. Furthermore, it was observed that the expression of CgCP reduces plant growth and delays the timing of floral transition. Quantitative RT-qPCR analysis of DELLA target genes showed that CgCP alters relative expression of several target genes, indicating that the DELLA proteins mediate transcriptional changes produced by CgCP expression. Analyses by fluorescence confocal microscopy showed that CgCP stabilizes DELLA proteins accumulation in the presence of gibberellic acid (GA) and that the DELLA proteins are also stabilized during TMV-Cg virus infections. Moreover, DELLA proteins negatively modulated defense transcript profiles during TMV-Cg infection. As a result, TMV-Cg accumulation was significantly reduced in the quadruple-DELLA mutant Arabidopsis plants compared to wild type plants.

**Conclusions:**

Taken together, these results demonstrate that CgCP negatively regulates the salicylic acid-mediated defense pathway by stabilizing the DELLA proteins during *Arabidopsis thaliana* viral infection, suggesting that CgCP alters the stability of DELLAs as a mechanism of negative modulation of antiviral defense responses.

## Background

During the co-evolutionary process established between plants and virus pathogens, plant viruses have evolved various means of altering host components to efficiently infect plants. Emerging evidence has shown that viruses disturb the defense regulatory pathways of plants by producing hormonal imbalances [1]. The role of hormones in the induction of disease had been suggested in earlier reports; however, these studies have not characterized the factors involved in hormonal imbalances [2]. Recently, some virus proteins have been shown to alter either or both the localization and activity of host components, which might influence the hormonal balance during compatible interactions [3],[4]. For example, *Rice dwarf virus* (RDV) P2 viral protein modifies a factor involved in the biosynthesis of gibberelic acid (GA) [3] and consequently there is a diminished accumulation of GA. The C2 protein of Geminivirus interferes with a regulatory component of the ubiquitination pathway [4], which is involved in the regulation of many hormonal responses [5]. Also, the TMV replicase protein was found to disrupt the localization of the auxin response regulator indole acetic acid IAA26/PAP1, thus altering the expression of auxin regulated genes [6],[7]. More recently, it was observed that TMV-Cg alters the function of a transcription factor involved in the balance of abscisic acid/ethylene signaling pathway [8].

Tobamoviruses have a single stranded RNA genome that encodes four well-characterized proteins: two proteins responsible for RNA-dependent RNA replication; another involved in cell to cell movement and, finally, a coat protein (CP) [9],[10]. Tobamovirus CPs are mainly associated with a structural function during virion assembly. However CP is also involved in several other aspects of virus infection [11]. For example, *Tobacco mosaic virus* CP (TMV CP) is required for systemic movement to distant plant tissues [12] and is found in viral replication complexes, suggesting that TMV CP may be required for efficient virus replication [13],[14]. Furthermore, TMV CP greatly affects the development of symptoms in viral infection [15] and confers heterologous interference to other virus [16].

Transgenic plants that accumulate TMV CP show a marked resistance towards the virus with a consequent delay in symptoms appearance [17]. Such phenomenon is known as coat protein-mediated resistance (CP-MR). CP transgenic plants inoculated with TMV-RNA overcome this resistance mechanism; which led Beachy to propose that CP blocks an early infection step [10]. Carr et al. [18] investigated a possible role of pathogenesis related proteins (PR proteins) in CP-MR but could not obtain positive evidence. In contrast, recent results obtained by our group showed that transgenic tobacco plants expressing TMV CP accumulate reduced mRNA levels of some salicylic acid (SA) responsive genes such as *pathogenesis-related (PR-1)* and *RDR1(RNA-dependent RNA polymerase 1)* when compared to WT (non-transgenic wild type) plants [19].

In many compatible plant-virus interactions, SA reduces viral replication and restricts cell-to-cell movement and systemic movement [20]–[22]. Studies in Arabidopsis leaves inoculated with RNA viruses showed that at least one third of the common sets of genes induced by viruses are associated with plant defense and stress responses [23]. Most importantly, the induction of many of these genes is compromised in SA-signaling mutants. This finding demonstrates that SA is involved in the viral-induced signaling that occurs during compatible virus interactions [24] and suggests that plant viruses have evolved strategies to evade the SA-induced defense during compatible interactions. Several studies have demonstrated that viral proteins can suppress SA signaling [25],[26], reinforcing the evasion hypothesis. The DELLA proteins are negative regulators of growth and in the presence of GA are degraded through the ubiquitin-proteasome pathway [27]. These proteins may also play a role as integrative hub in hormone cross-talk [28]. Furthermore, they are also known to promote susceptibility to biotrophic bacterial pathogens by repressing SA defense response in *Arabidopsis thaliana*[29]. Recently, several reports have shown that viruses alter hormone biosynthesis and signaling [1]. However, the role of DELLA proteins in the modulation of signaling events during virus infection has not been characterized yet.

To extend the analysis of the role of Tobamovirus coat proteins during compatible virus interactions, we conducted studies to determine if CP from TMV-Cg is capable of modulating defense responses in *Arabidopsis thaliana*. TMV-Cg is a Tobamovirus that infects Arabidopsis [30]. For this experiment, we used transgenic plants expressing CgCP under an inducible promoter [31]. The transgenic expression of CgCP stabilized DELLAs proteins and suppressed the expression of a set of SA-responsive genes. In the present study, we determined the role of DELLAs proteins in the modulation of signaling events during TMV-Cg infection. We demonstrated that during TMV-Cg infection CgCP stabilizes DELLAs proteins and this stabilization results in the modulation of SA-defense signaling pathway.

## Results

### CgCP expression downregulates the expression of SA-responsive genes in Arabidopsis but does not alter SA or JA levels

**Figure 1:**
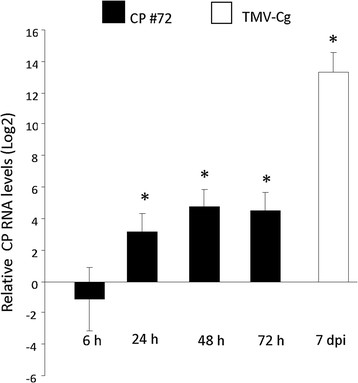
**Accumulation of CgCP in inducible CP#72 transgenic line and TMV-Cg-infected non- transgenic Col-0 plants compared to constitutive CgCP expressing line CP#71.** Relative expression levels of Cg CP were determined by qRT-PCR. The relative expression levels of Cg CP in the inducible CP#72 transgenic line at different time points after the induction (6, 24, 48 and 72 h) are shown. TMV-Cg infected plants samples were taken at 7 dpi. In all cases the CgCP accumulation level was compared to the level of CgCP expression of a constitutive transgenic line (CP#71). The expression level in CP#71 line was arbitrarily set to one (Log_2_(1) = 0). Asterisks indicate statistically significant differences (* = P values < 0.05).

To study the role of CgCP expression during viral defense signaling responses, we employed an Arabidopsis transgenic line (CP#72) harboring the CgCP gene that is regulated by an inducible promoter [31]. This chemically inducible gene expression system employs methoxyfenozide (MOF) as the inducer [31],[32]. The CP#72 transgenic plants were grown to stage 1.08 [33] (approximately 20 days after germination) to establish the CgCP expression level. Then, the plants were treated with MOF and samples were collected at different time points after induction. RT-qPCR was used to compare the level of the CgCP mRNA from CP#72 with the level of a constitutive transgenic line expressing TMV-Cg CP under the *Cauliflower mosaic virus* (CaMV) 35S promoter (CP#71). The CgCP accumulation level was also determined in the WT-Col-0 (non-transgenic) TMV-Cg infected plants. Twenty-four hours post CgCP induction, the inducible line (CP#72) showed higher levels of CP transcripts than the line that constitutively expresses CgCP (CP#71) (Figure 1). The maximum level of accumulation was reached at 48 h and was maintained at 72 h post-induction (Figure 1). Nevertheless, the level was lower than the level reached 7 days post infection (dpi) in the TMV-Cg infected plants (Figure 1). The temporal pattern of CgCP accumulation reported here was similar to that reported by Koo et al. [31].

In a previous work, we demonstrated that *N. tabacum* transgenic plants expressing TMV CP accumulate reduced levels of *PR-1* and *RDR-1* transcripts [19]. Based on those findings, we selected a group of SA-responsive genes involved in antiviral defense signaling in order to quantify their transcript levels in the CP#72 transgenic line: *RDR1*; *AOX1A* and *WRKY70*. RDR1 is a component of RNA silencing machinery [34]. AOX1A is the terminal oxidase of the cyanide-resistant alternative respiratory pathway in plants, which is also implicated in virus resistance [21]. Finally, WRKY70 was chosen based on its well-known regulatory role in SA signaling pathway [35].

**Figure 2:**
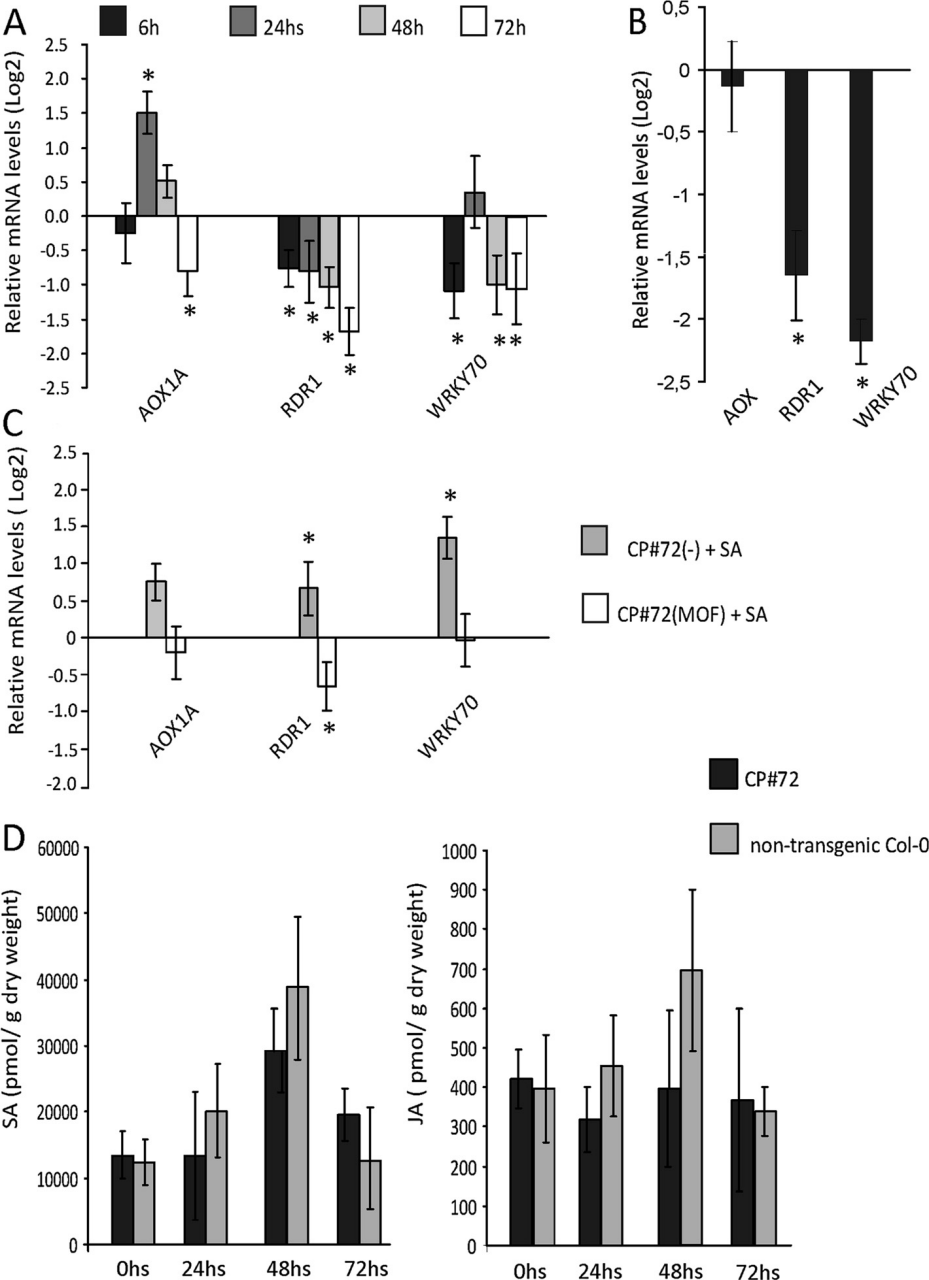
**CgCP expression downregulates transcriptional levels of SA-responsive genes, but it does not alter endogenous SA or JA levels. A)** Relative mRNA levels of *AOX1A*, *RDR1* and *WRKY70* genes after CgCP induction in CP#72(MOF) plants (MOF treated) over a 72 h time course. Non-induced CP#72(−) (water treated) were used as reference controls and the expression level was arbitrarily set to one (Log_2_ (1) = 0). **B)** Relative mRNA levels in constitutively expressing CP #71 line compared to WT Col-0 plants at 1.08 developmental stage. The expression level in WT line was arbitrarily set to one (Log_2_(1) = 0). **C)** Relative mRNA levels in CP#72 line after SA treatment. CP#72(−) (water treated) or MOF-treated CP#72 (MOF) plants at 48 h post induction were sprayed either with 0.5 mM SA (+SA) or water (−). The expression levels were referenced to non-induced CP#72(−) plants (water treated) and arbitrarily set to one (Log_2_(1) = 0). **A)**, **B)** and **C)** Relative transcript levels were determined by RT-qPCR. Data correspond to the mean ± standard error of four biological replicates. Asterisks indicate statistically significant differences (* = P values < 0.05). **D)** JA and SA accumulation level determined by HPLC in induced CP#72 and non-transgenic Col-0 plants at 0, 24, 48 and 72 h post induction with MOF. Data at 0 h correspond to untreated plants. The means of four biological replicates ± SE are shown.

A reduction of *RDR1* and *WRKY70* mRNA levels was observed in MOF-treated CP#72 plants compared to water-treated CP #72 plants at 48 and 72 h post induction, while a reduction of *AOX1A* mRNA levels was detected only 72 h post CgCP induction (Figure 2A). In parallel, we quantified the same set of genes in the CP#71 independent transgenic line (Figure 2B). *RDR1* and *WRKY70* also showed reduced expression levels in this independent transgenic line when compared to the non-transgenic plants. However, *AOX1A* remained unaltered in the CP#71 transgenic line.

An additional control experiment was performed to exclude that the transcriptional changes observed in the CP#72 transgenic lines were because of the MOF treatment. We determined the level of expression of these transcripts in WT- Col-0 plants treated either with water or MOF (48 h after MOF). The results from this experiment showed no statistically significant differences between both treatments, confirming that all the expression changes are exclusively due to CgCP expression (Additional file 1).

Next, we analyzed the expression levels of *WRKY70*, *AOX1A* and *RDR1* in response to CgCP induction under SA hormone treatment (Figure 2C). For this purpose, CP#72 transgenic plants were initially treated with MOF or water and subsequently (48 h later) sprayed with 0.5 mM SA or water. Gene expression levels were analyzed 24 h after hormone treatment using as reference untreated CP#72 plants (no MOF and no SA) (CP#72 control). As expected, the levels of *WRKY70*, *AOX1A* and *RDR1* were increased in the control plants (exposed to water) after SA treatment (CP#72(−) + SA). However, the SA treatment did not increase gene expression in MOF -induced CP#72 plants (CP#72(MOF) + SA), when compared to water-treated CP#72 control plants (Figure 2C). Thus, these results indicate that CgCP negatively modulates the *WRKY70*, *AOX1A* and *RDR1* transcript levels after SA treatment.

As CgCP altered the expression of genes involved in SA signaling, we next quantified the levels of SA and jasmonic acid (JA) in CP#72 transgenic plants and WT-Col-0 treated with MOF. JA was selected because of its antagonistic role to SA. After MOF treatment, no statistical differences were observed in SA or in JA levels between CP#72 and WT plants throughout the study, not even at 0 h (non-induced plants) (Figure 2D). This finding is in agreement with our previous results in TMV-infected *Nicotiana tabacum*[19]. Therefore, these experiments demonstrate that CgCP modulates the SA signaling without altering SA or JA basal levels.

### CgCP expression reduces plant growth and delays the timing of floral transition

Recent evidence showed that plant viruses can interfere with hormone synthesis and signaling and, as a result, plant development is altered [1]. To study whether CgCP alters *Arabidopsis thaliana* normal development, we analyzed the phenotype of CgCP transgenic plants in detail.

**Figure 3:**
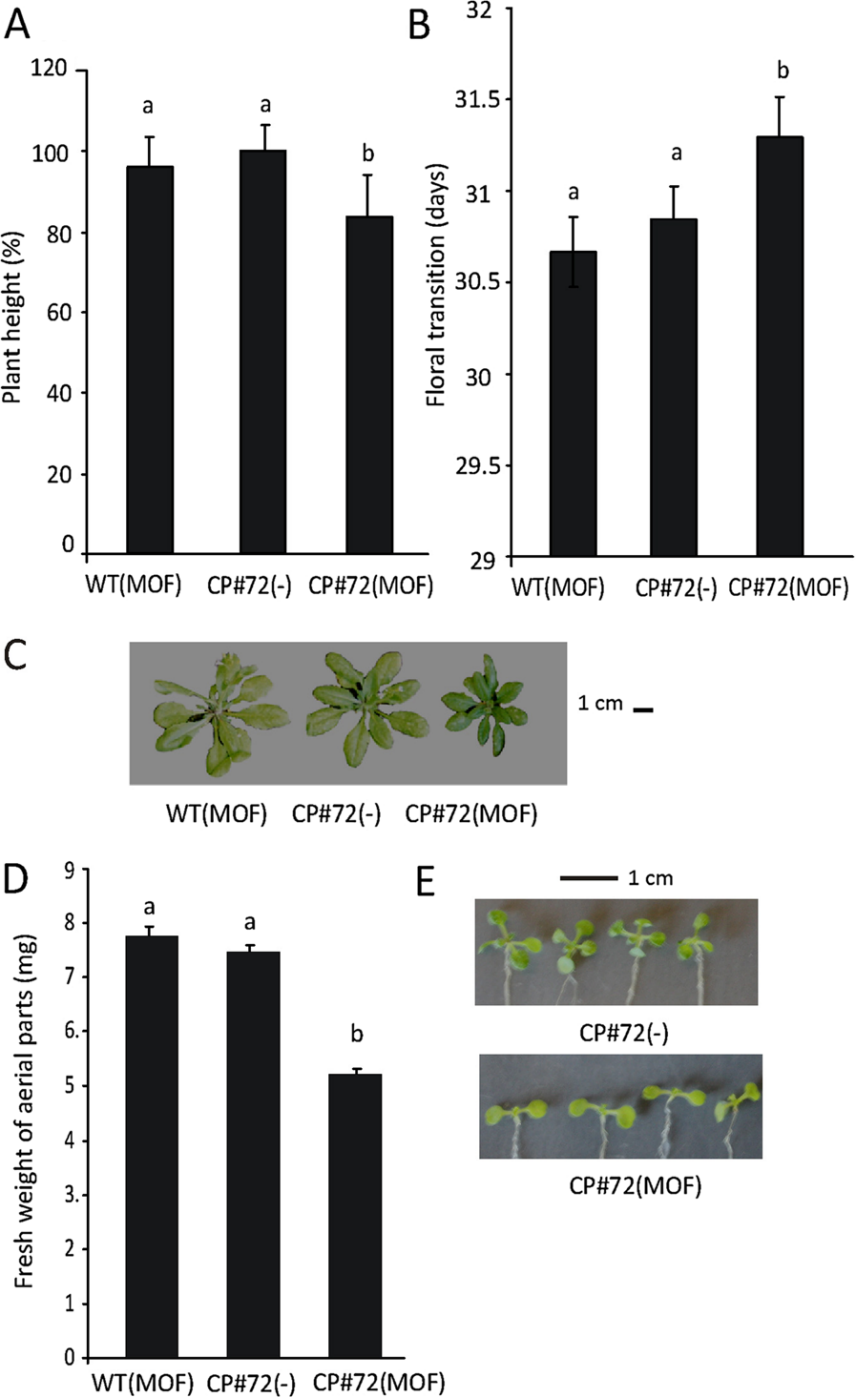
**CgCP expression reduces plant growth and delays the timing of floral transition.** Measurements were taken in MOF-treated WT Col-0 and MOF-treated CP#72 plants. Data were compared to non-induced CP#72(−) plants (water treated). **A)** Bar graph showing plant height (6-weeks-old) and **B)** floral transition (number of days to first flower). Each column represents the mean of 25 plants ± SE. **C)** Representative WT Col-0 and CP#72 transgenic 6-week-old plants grown in presence of MOF (MOF) or water (−). **D)** Bar graph showing the average fresh weight of Col-0 (WT) and CP#72 transgenic 10-day-old seedlings grown in presence of MOF (MOF) or water (−) for 7 days. Each column represents the mean value of 18 plants ± SE. **E)** Representative 10-day-old CP#72 transgenic plants grown in presence of MOF (MOF) or water (−) for 7 days. Similar results were obtained in two independent experiments for all measurements. Different letters above the bars represent the significant differences among samples, p-values ≤ 0.05.

The MOF-induced CP#72 plants showed a reduction in plant height (Figure 3A) and a delay in flowering time (Figure 3B) when compared to CP#72 plants that were treated with water. As another control, non-transgenic Col-0 plants were treated with MOF. No statistical differences were detected when these controls were compared with the CP#72 plants that were treated with water. Thus, the expression of CgCP was the main cause of the delay in the height and timing of floral transition (Figure 3A, B and C). We also evaluated the phenotype of CP#71 transgenic plants (Additional file 2). Similarly to that observed in CP#72 transgenic plants treated with MOF, CP#71 showed a reduction in plant height (Additional file 2) and a delay in flowering time (Additional file 2) when compared to non-transgenic Col-0 plants. These developmental changes had not been reported in Koo et al.’s work (2004), maybe due to the differences in the used experimental procedures. In the experiments carried out by Koo et al. using the CP#72 transgenic line, MOF treatment of the CP#72 line was performed at a later developmental stage (five/six weeks of age), when inflorescences have already emerged [33]. For this reason, CP accumulation had no effect on the timing of floral transition. Accordingly, when we analyzed the seedling development, in CP#72 transgenic line, we detected striking reductions of growth in the aerial parts of the MOF-induced CP#72 transgenic line in comparison with the seedlings that were treated with water (Figures 3D and E). For this analysis, we also used non-transgenic Col-0 seedling plants treated with MOF as controls (wild type plants, WT). As shown in Additional file 3, no differences were observed in the growth of MOF-treated WT Col-0 plants in comparison with water-treated non-transgenic Col-0 plants. Therefore, CgCP altered the normal development of Arabidopsis seedlings and adult plants but only when CgCP is expressed during the early stages of development. The effect of CgCP expression on development and hormone signaling pathways suggests that CgCP could be altering a fundamental component involved in the regulation of growth and hormone cross-talk. The DELLA proteins are central to both processes [28],[36],[37]; therefore, these proteins could play a role in the phenotypes observed during CgCP expression. Together, this data prompted us to analyze the role of DELLA proteins during CgCP expression.

### CgCP increases the expression of DELLA target genes and stabilizes DELLA protein accumulation

To explore whether DELLAs play a role in the changes observed during CgCP expression, we analyzed the mRNA transcript levels of a set of DELLA-responsive genes (Zentella et al., 2007). *UBIQUITIN- CONJUGATIN ENZYME 17* (*UBC17*), *IQ-DOMAIN 22* (*IQD22*), *GA INSENSITIVE DWARF1B* (*ATGID1b*) and *BASIC HELIX –LOOP HELIX 137 (bHLH137)* are transcripts which are downregulated by GA and upregulated by DELLA protein stabilization in seedlings plants [38]. To study the transcript levels of this set of genes in rosette leaves, we quantified their expression in Arabidosis cv Ler quadruple-DELLA mutants (plants lacking four DELLA genes: *RGA*, *GAI*, *RGL1*, *RGL2*) [27] and also in Ler *gai-1* mutants (gibberellin-insensitive plants due to GAI-constitutive stabilization) [39]. For this experiment, we collected rosette leaf tissue at 1.08 developmental stage. *UBC17* and *bHLH 137* transcript levels were statistically increased in the *gai-1* plants compared to the non-mutant Ler plants (Additional file 4). On the other hand, *UBC17*, *IQD22*, *ATGID1b,* transcript levels were statistically reduced in the quadruple-DELLA mutants compared to the non-mutant Ler plants (Additional file 4).

**Figure 4:**
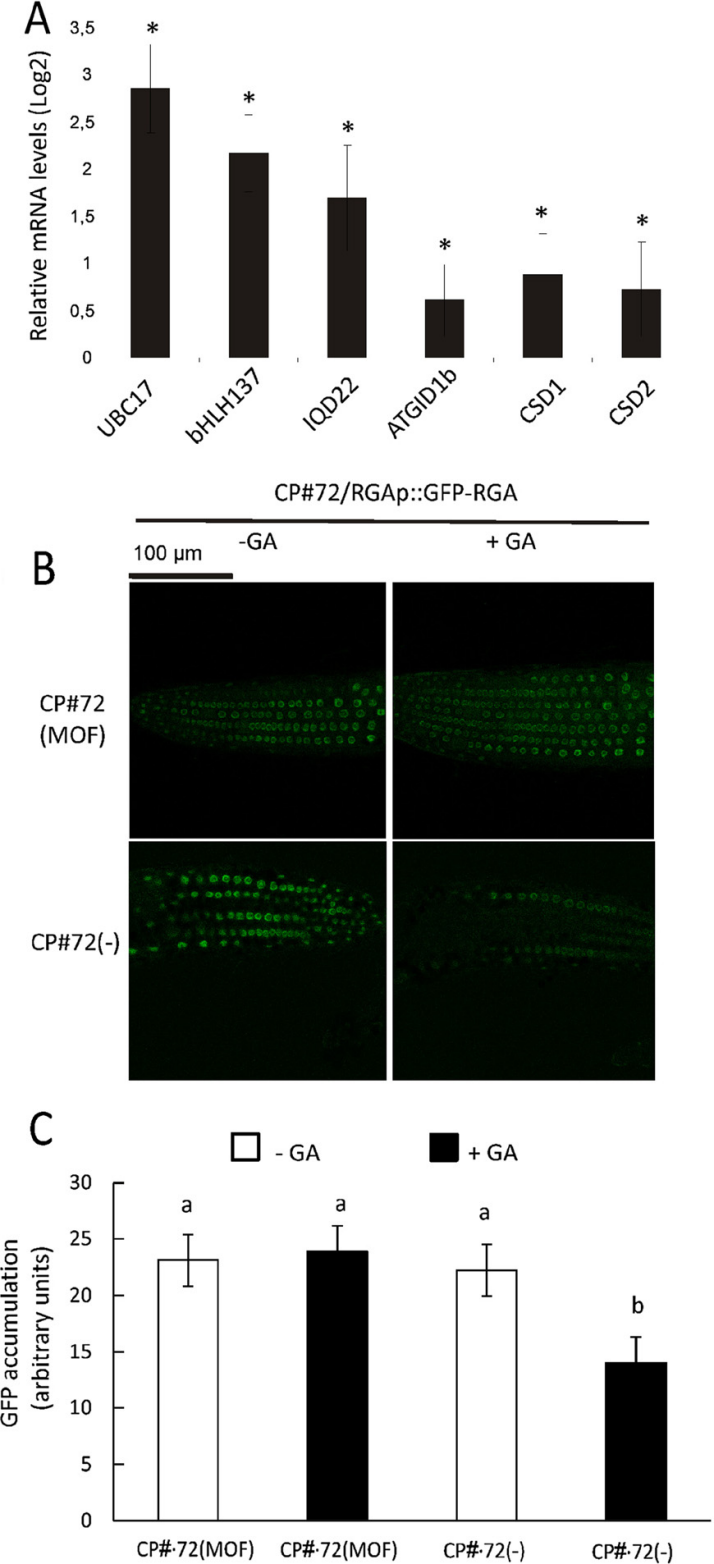
**CgCP expression increases the expression of DELLA targets and stabilizes DELLA accumulation A).** Relative mRNA levels of DELLA target genes *UBC17*, *bHLH137, IQD22*, *ATGID1b, CSD1* and *CSD2* were determined by RT-qPCR in induced CP#72 plants (72 h after MOF treatment) compared to water-treated CP#72(−) plants. The expression level in CP#72(−) plants was arbitrarily set to one (Log_2_(1) = 0). The means ± SE of four biological replicates are shown. Asterisks indicate statistically significant differences (* = P values < 0.05). **B)** GFP fluorescence observed in induced (MOF) and water- treated (−) CP#72/RGAp::GFP-RGA primary seedling roots. After 3 days of induction (MOF treatment), seedlings were subsequently treated with 10 μMGA_3_ (+GA) or water (−GA) for 1 h. The experiment was repeated twice with similar results. **C)** The bar graph shows the levels of accumulation of GFP present in the nuclei of Arabidopsis cells. Quantitative analysis was performed as is explained in Methods. Different letters above the bars represent the significant differences among samples, p-values ≤ 0.05.

Next, to determine the effect of CgCP on the transcription level of these genes, we quantified their transcription after CgCP induction. The MOF-induced CP#72 (72 h after MOF exposure) plants accumulated higher levels of all the DELLA-target genes analyzed compared to the non-induced CP#72 plants (Figure 3A). In addition, we evaluated the transcription level of two other DELLA regulated genes. The transcription levels of Cu/Zn superoxide dismutases (Cu/ Zn SOD), *CSD1* and *CSD2*, are positively modulated by DELLA proteins [36]. As shown in Figure 4A, both *CSD1* and *CSD2* transcript levels were upregulated by CgCP; which suggests that the modulation of gene expression observed during CgCP expression might be regulated by DELLA proteins.

To determine whether the DELLA proteins are stabilized by CgCP expression, we analyzed protein accumulation after CgCP induction. For this purpose, we crossed a transgenic line expressing RGA, one of the five DELLA proteins, fused to GFP (pRGA::GFP-RGA) [40] with CP#72 line obtaining CP#72/pRGA::GFP-RGA. By using the homozygous sibling carrying both transgenes, we evaluated the levels of RGA-GFP in MOF- or water-treated seedlings in the presence of gibberellic acid (GA_3_) (10 μM). RGA-GFP can be easily detected by fluorescence confocal microscopy at seedling stages [29]. As expected, RGA-GFP was degraded by GA treatment (Figure 4B) in the non-induced CP#72/pRGA::GFP-RGA seedlings. However, CgCP expression reduced GA-mediated degradation of RGA-GFP in Arabidopsis seedlings (Figure 4B).

With the aim of quantify the amount of GFP-RGA protein that was accumulated in the nuclei of Arabidopsis cells, the GFP-RGA fluorescence intensity of twenty random nuclei from five different plants for each treatment were analyzed using the ImageJ Software (for more detail, see Methods). No statistically significant differences were detected when GFP-RGA fluorescence was compared between CP#72/RGA::GFP-RGA plants treated with MOF and CP#72/RGA::GFP-RGA plants treated with water, prior the addition of GA_3_. After addition of GA_3_, GFP-RGA fluorescence was significantly reduced in nuclei of CP#72/RGA::GFP-RGA transgenic plants treated with water (Figure 4C and Additional file 5). In contrast in CP#72/RGA::GFP-RGA MOF-treated plants, the GFP-RGA fluorescence was not reduced after the addition of GA_3_.(10 μM ) (Figure 4C and Additional file 5). Taken together, these experiments demonstrate that CgCP stabilizes DELLA proteins and, as a consequence, alters gene expression profiles.

### CgCP suppresses a set of defense genes that are regulated by DELLA proteins

**Figure 5:**
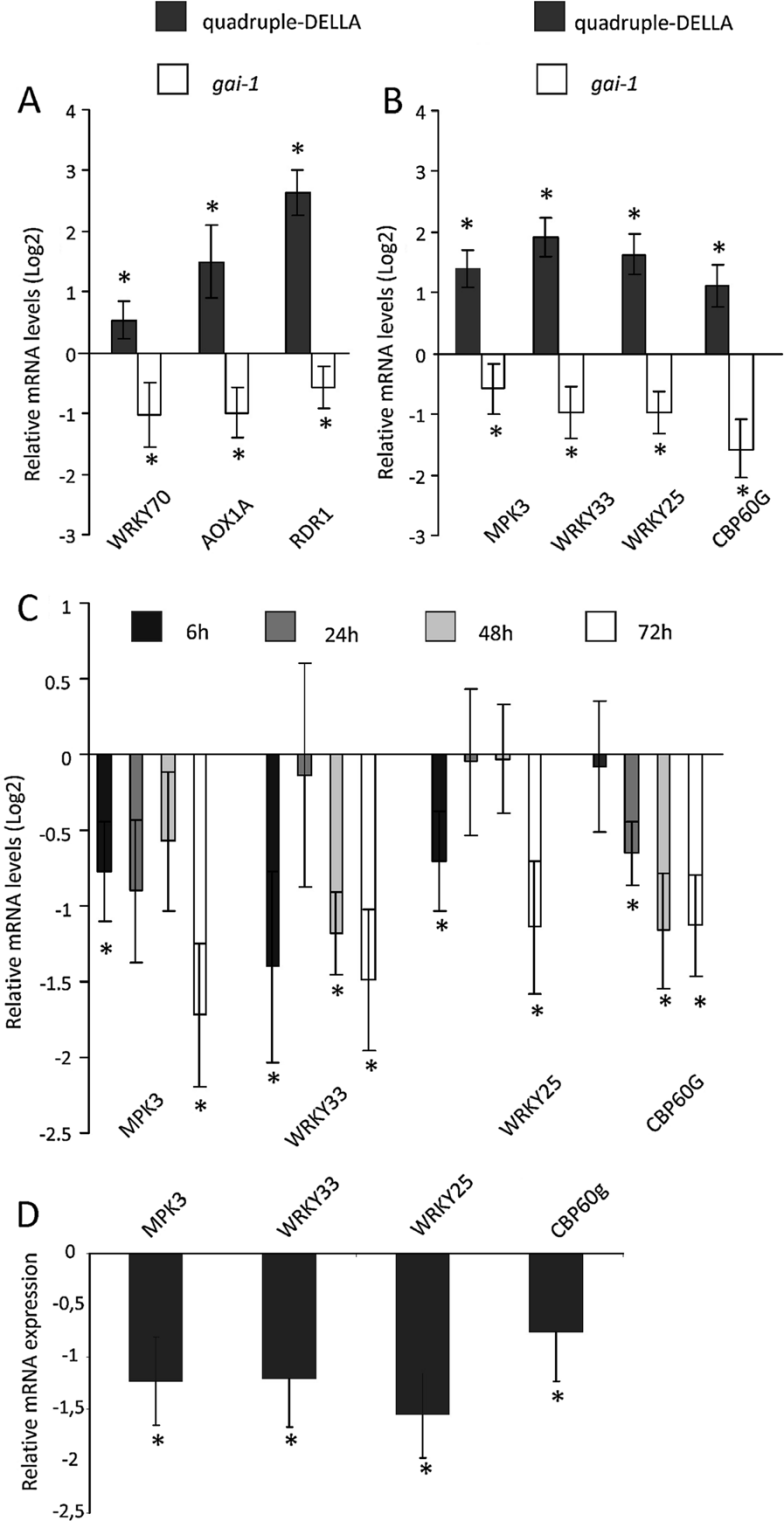
**CgCP expression suppresses a set of defense genes regulated by DELLA proteins. A)** Relative mRNA levels determined by RT-qPCR of SA-responsive genes (*WRKY70*, *AOX1A* and *RDR1*) and **B)** DELLA related defense genes (*MPK3*, *WRKY33*, *WRKY25* and *CBP60g*) in *gai-1* and quadruple-DELLA mutants compared to WT-Ler plants. The expression level in WT-Ler plants was arbitrarily set to one (Log_2_(1) = 0). **C)** Relative mRNA levels of DELLA related defense genes determined by RT-qPCR in induced CP#72 plants (MOF treated) compared to water-treated CP#72(−)control plants over a 72 h time course. The expression level in CP#72(−) plants was arbitrarily set to one (Log_2_(1) = 0). **D)** Expression of *CBP60g*, *MPK3*, *WRKY25* and *WRKY33* in CP#71 transgenic line. Relative mRNA levels of individual genes in CP#/71 plants were calculated in comparison to WT-Col-0 plants. Expression level in WT-Col-0 plants was arbitrarily set to one (Log_2_(1) = 0). **A)**, **B)**, **C)** and **D)** The means ± SE of four biological replicates are shown. Asterisks indicate statistically significant differences (* = P values < 0.05).

CgCP expression downregulated a set of SA-responsive genes (*WRKY70*, *AOX1A* and *RDR1)* and also stabilized the accumulation of DELLA proteins. Hence, we analyzed the expression levels of these transcripts in quadruple-DELLA and *gai-1* mutants and, as expected, found that *WRKY70*, *AOX1A* and *RDR1* were upregulated in the quadruple-DELLA mutants but downregulated in the *gai-1* mutants. Wild type Arabidopsis Ler plants were used as reference controls for these experiments (Figure 5A). The regulation of *RDR1* by DELLA proteins is particularly interesting since this gene is essential for maintaining basal resistance to several RNA viruses including TMV-Cg [34].

SA is known to induce multiple virus resistance mechanisms [41],[42] and *RDR1* is only one of the components involved in this mechanism. We proceeded to determine whether the DELLA protein stabilization by CgCP induction alters the expression of other important genes involved in SA-induced resistance. For this purpose, we included a larger set of candidates. The selection was based on the analysis of a microarray study performed between Arabidopsis *gai-1* mutants and non- mutant (WT) plants treated with flg22 (GEO accession: GSE17464). Data were gathered from Genevestigator data base [43] (Additional file 6). This new set of candidate genes, which are involved in SA biosynthesis, innate immune signaling and stress response, showed reduced expression levels in the *gai-1* mutant plants after flg22 (*gai-1* + flg22) treatment compared to the non-mutant plants treated with flg22 (WT + flg22) (Additional file 6). *CBP60G* encodes a calmodulin binding protein that plays a role in SA accumulation and SA signaling [44]; *MPK3* encodes a MAPKs activated by pathogen infections [45]; *WRKY25* and *WRKY33* respond to stress and increase expression after SA treatment [46],[47]. By RT-qPCR, the transcription levels of these defense genes were quantified in the quadruple-DELLA mutants as well as in *gai-1* mutants and these levels were compared to those of the WT-Ler control plants (Figure 5B). A higher level of expression of all these genes was detected in the quadruple-DELLA mutants compared to that of the WT-Ler plants. Instead, the *gai-1* mutants showed reduced levels of these transcripts.

Next, we studied the expression of these genes in either MOF-induced or water-treated CP#72 plants at 6, 24, 48 and 72 h after CgCP induction (Figure 5C). Again, reduced expression levels were observed for all four genes in the CP#72 plants after MOF treatment when compared to the CP#72 plants treated with water. A similar downregulation of this set of genes was observed in a CP#71 independent transgenic line that constitutively expresses CgCP when compared to the non-transgenic Col-0 plants (Figure 5D). Taken together, the expression profiles observed after CgCP expression are similar to those observed in the *gai*-1 mutants and opposed to the DELLA-quadruple mutants. Thus, these findings strongly suggest that CgCP expression is attenuating the expression of defense genes through the stabilization of DELLA proteins.

### TMV-Cg infection stabilizes DELLA protein accumulation

**Figure 6:**
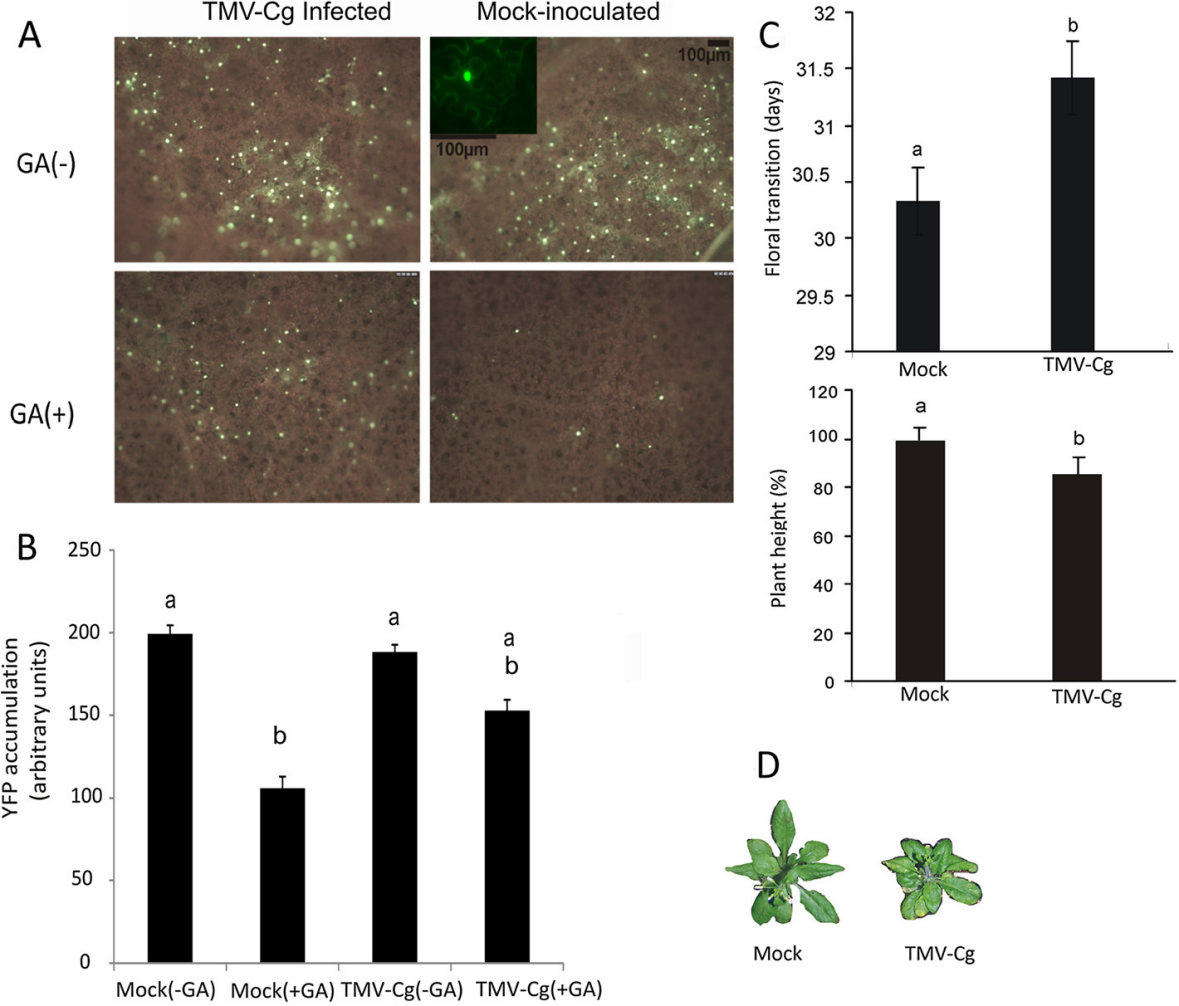
**DELLA proteins are stabilized during TMV-Cg infection and reduce plant growth and delay the timing of floral transition. A)** Accumulation of YFP-GAI in the nucleus observed using fluorescence microscope. TMV-Cg infected *N. benthamiana* leaves and mock-inoculated *N. benthamiana* leaves were agroinfiltrated with a construction carrying YFP-GAI at 6 dpi. At 72 h post-agroinfiltration leaves were sprayed with 100uMGA_3_ (GA+) or water (GA-) and subsequently observed an hour later. This experiment was repeated twice with similar results. **B)** The bar graph shows the levels of accumulation of YFP present in the nuclei of N. benthamiana cells. Quantitative analysis was performed as is explained in Methods. **C)** The third true leave of non-transgenic Col-0 (grown to stage 1.08) plants were inoculated with TMV-Cg virus. The floral transition and plant growth (plant height at six weeks) were determined in TMV-Cg infected plants as compared to mock-inoculated plants. **D)** Representative 6-week-old mock-inoculated non- transgenic Col-0 plants and infected non-transgenic Col-0 plants. **B)** and **C)** Different letters above the bars represent the significant differences among samples, p-values ≤ 0.05.

Based on the results obtained, we decided to determine whether the CgCP effect on DELLA stabilization is also produced during TMV-Cg infection. For this experiment, *Nicotiana benthamiana* plants were inoculated with TMV-Cg virus or buffer (mock-inoculated). Later on (6 days), upper leaves plants were agroinfiltrated with YFP-GAI-expression construct [48]. *N. benthamiana* was selected because this plant allows a good expression of YFP-GAI fusion and easy detection using a fluorescence microscope [4]. Three days post-agroinfiltration, YFP-GAI accumulation was detected predominantly in nuclei of both TMV-Cg-infected *N. benthamiana* plants and virus mock-inoculated plants (see Figure 6A inset). Leaf explant of both set of plants were treated with GA_3_ (100 μM) solution for an hour and were subsequently observed on a fluorescent microscope. As expected, the fluorescence decreased after GA treatment in the mock-inoculated plants. A reduction of YFP-GAI fluorescence was also observed in the TMV-Cg infected plants but to a lesser extent, showing a detectable difference when compared to the mock-infected plants (Figure 6A). To quantify the amount of YFP-GAI protein that was accumulated in the nuclei of *N. benthamiana* cells, the YFP-GAI fluorescence intensity of twenty random nuclei from five different plants for each treatment were analyzed using the ImageJ Software (for more detail see Methods section). The intensity of YFP-GAI fluorescence accumulated in nuclei of cells of TMV-Cg infected plants was not statistically different from the intensity of YFP-GAI fluorescence detected in nuclei of cells of mock-inoculated plants. However, it was observed a statistically significant reduction of intensity of YFP-GAI fluorescence in nuclei of mock-inoculated plants treated with GA, compared to the YFP-GAI fluoerescence accumulation in nuclei of cells of mock-inoculated plants, previously to the GA treatment (Figure 6B). As is shown in Additional file 7, the amount of nuclei in which YFP-GAI protein was detected and the YFP-GAI fluorescence level, were reduced in mock-inoculated plants treated with GA (Additional file 7B). Alternatively, when TMV-Cg infected plants were treated with GA, the YFP-GAI accumulation detected in nuclei was not significantly reduced compared to the YFP-GAI accumulation detected in the nuclei of infected plants previous to GA treatment (Figure 6B and Additional file 7D). Thus, these results clearly indicate that TMV-Cg virus delays GA-mediated DELLAs degradation.

Furthermore, we analyzed the symptoms produced during TMV-Cg virus infection in *Arabidopsis thaliana* plants. In concordance with the stabilization of DELLA proteins observed in *N. benthamiana* during TMV-Cg infection, Arabidopsis plants infected with TMV-Cg virus display a reduction in height and a delay in floral transition (Figure 6C and D).

### DELLA proteins modulate transcript profiles during TMV-Cg infection

**Figure 7:**
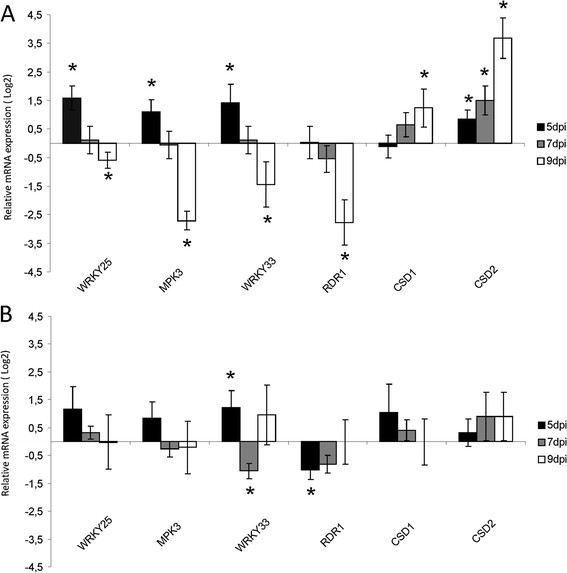
**DELLA proteins modulate transcript profiling during TMV-Cg infection. A)** Relative mRNA levels of *WRKY25*, *WRKY33*, *MPK3*, *RDR1*, *CSD1, and CSD2* were determined by RT-qPCR in TMV-Cg infected WT-Ler plants compared to mock-infected WT Ler plants at 5, 7 and 9 dpi. Expression level in mock inoculated WT plants was arbitrarily set to one (Log_2_(1) = 0). **B)** Expression levels of *WRKY25*, *WRKY33*, *MPK3, RDR1, CSD1* and *CSD2*, in quadruple-DELLA mutant infected plants compared to non-infected quadruple-DELLA mutant plants at 5, 7 and 9 dpi. Expression level in mock inoculated quadruple-DELLA plants was arbitrarily set to one (Log_2_(1) = 0). **A)** and **B)** The means ± SE of four biological replicates are shown. This experiment was repeated twice with similar results.

The stabilization of the DELLA proteins during TMV-Cg infection led us to hypothesis that these proteins could be involved in the modulation of transcript profile during such infection. Therefore, we characterized the role of the DELLA proteins on the modulation of DELLA-regulated genes during virus infection. For this purpose, WT Arabidopsis plants were infected with TMV-Cg virus and samples were taken at 5, 7 and 9 days post infection to determine the transcriptional levels of a set of DELLA-regulated genes by RT-qPCR. As shown in Figure 7A, *WRKY25*, *WRKY33*, *MPK3* transcript levels were increased (almost two folds or more) in the TMV-Cg infected WT plants compared to the mock-inoculated WT plants at 5 dpi. However, at 7 dpi no differences of expression for these transcripts were detected between the infected and mock-inoculated plants (Figure 7A). At 9 dpi, the level of those transcripts was reduced in the infected WT plants compared to the mock -inoculated WT plants (Figure 7A). The expression level of *RDR1* did not change in the TMV-Cg infected WT plants with respect to the mock-inoculated WT plants at 5 and 7 dpi, but decreased significantly at 9 dpi (Figure 7A). The reduced abundance of these transcripts levels at 9 dpi was in agreement with the decreased abundance previously observed during CgCP transgenic expression.

In parallel, the expression of two other DELLA targets, *CSD1* and *CSD2*, were determined in WT plants at the same point times post infection. *CSD1* showed no change at 5 dpi but its abundance was clearly augmented at 7 and 9 dpi. Instead, *CSD2* showed a slight increase starting at 5 dpi and a higher increment at 7 and 9 dpi (Figure 7A). Likewise, *CSD1* and *CSD2* transcript levels were reported to increase by DELLAs stabilization [36]. Therefore, these results show that DELLA-target transcripts are altered during the course of TMV-Cg infection, suggesting that the DELLA proteins may be involved in the defense response to TMV- Cg.

To further study the role of DELLA proteins over the modulation of transcript profile, quadruple-DELLA mutant plants were infected with TMV-Cg virus. *WRKY25* and *MPK3, CSD1* and *CSD2* transcripts levels were not altered in TMV-Cg infected quadruple-DELLA mutant plants compared to the mock-inoculated quadruple DELLA mutant plants at 5 and 7 dpi, while *WRKY33* and *RDR1* altered their transcriptional levels at 5 and 7 dpi (Figure 7B). Contrary to what was observed in WT plants, none of the genes under studied alters its expression at 9 dpi in the TMV-Cg infected quadruple-DELLA plants compared to the non-infected quadruple DELLA mutant plants (Figure 7B). These results show that DELLA proteins are involved in the modulation of transcript profile during TMV-Cg infection.

### DELLA protein stabilization attenuates the defense response to TMV-Cg infection

**Figure 8:**
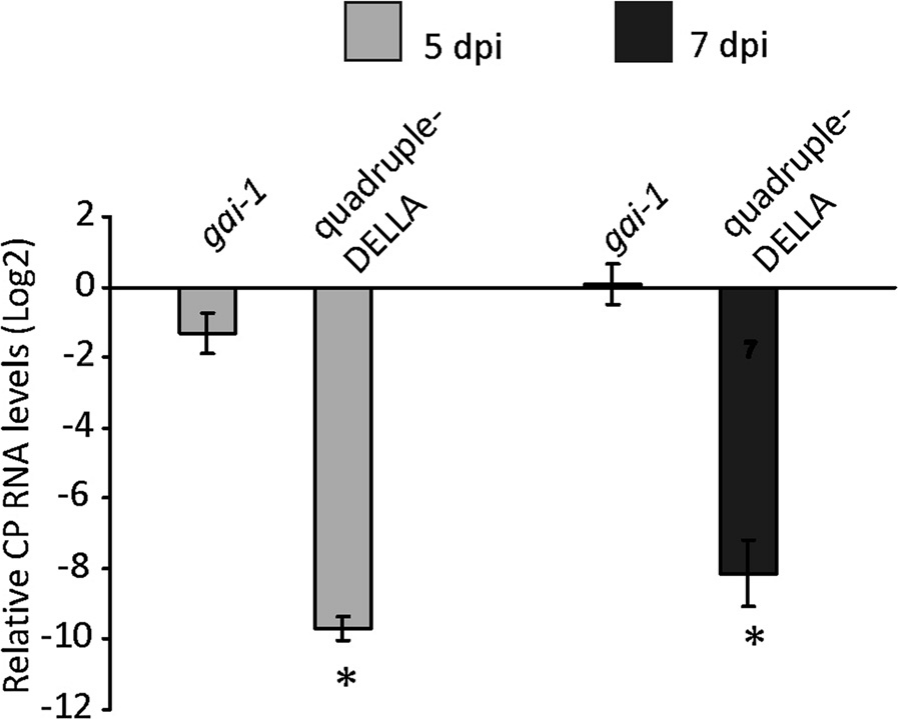
**DELLA proteins reduce the level of TMV-Cg CP RNA accumulation.** Relative CgCP RNA levels determined by RT-qPCR in TMV-Cg infected *gai-1* and quadruple-DELLA mutants at 5 and 7 dpi compared to TMV-Cg infected WT-Ler plants. The means ± SE of 7 biological replicates are shown for each treatment. Asterisks indicate statistically significant differences (* = P values < 0.05).

To determine whether the role of DELLA proteins on SA-mediated pathway has any effect on TMV-Cg infection level, we analyzed the level of CP accumulation in quadruple-DELLA and *gai-1* mutants compared to the CP accumulation in WT Ler plants. These analyses were performed by RT-qPCR at 5 and 7 dpi. A reduced accumulation of TMV-CgCP (more than 100-fold reduction) in the quadruple-DELLA mutants was observed both at 5 and 7 dpi (Figure 8). However, no differences were detected in CP accumulation between the *gai-1*-mutant plants and the WT Ler plants (Figure 8). In addition, we analyzed the replicase transcript accumulation in quadruple-DELLA mutant compared to the non-mutant Ler plants. Similarly, we observed a reduced accumulation of TMV-Cg replicase in quadruple-DELLA mutants compared to the non-mutant Ler plants (Additional file 8). These results suggest that the modulation of defense pathways by DELLA proteins could be responsible for the reduction of viral accumulation in the non-mutant plants versus the quadruple-DELLA mutants.

## Discussion

### CgCP downregulates the SA defense response

Compatible host-virus interactions result in systemic infections that trigger broad changes in gene expression [49]. To study the contribution of CgCP to the alteration of host gene expression, we employed transgenic plants that express the CgCP under an inducible promoter. Using this system, we demonstrated that CgCP reduces the expression of a set of SA-responsive genes (Figure 2A and B). Also, we studied the expression of these genes in a transgenic plant that express the CgCP under a constitutive promoter. In both transgenic lines we observed a reduction in transcript accumulation of the genes evaluated, with the exception of *AOX1A.* While the expression level of *AOX1A* was induced in CP#72 plants at 24 h post induction and reduced at 72 h post induction, it remained unaltered in the CP#71 transgenic line, which constitutively expresses CgCP. By contrast, all the other genes behaved similarly in both plant lines. One possible explanation could be that CgCP only causes a transient modulation of *AOX1A* expression. Alternatively, it could be that the expression level of AOX1A is altered only when CgCP accumulation reaches higher levels than the observed in the constitutive CP#71 transgenic line.

SA induces the expression of SA-responsive genes in CP#72 plants in the absence of MOF; however, in the presence of MOF, when CgCP is being expressed, SA fails to induce the expression of these set of genes (Figure 2C). These results are in concordance with previous observations made by our group using transgenic tobacco plants that constitutively express TMV CP [19].

A previous study that analyzes the Arabidopsis-Tobamovirus interactions showed that TMV downregulates the expression of SA-signaling components in systemically infected tissues [25]. In the present study, we observed that CgCP reduces the expression of a set of SA-responsive genes in a context of unchanged SA level. These results suggest that CgCP may negatively modulate the SA-sensing/signaling pathway by interfering with host components involved in hormone cross-talk. Recently, several studies have shown that plant viral proteins suppress the action of defense signaling components [25],[26]. In particular, P6 of CaMV acts as a pathogenicity effector and reduces the expression of SA-responsive genes [26]. In fact, P6 modifies NONEXPRESSOR OF PATHOGENESIS-RELATED1 (NPR1) and, as a consequence, reduces the expression of SA-responsive genes, but increases the expression of JA-dependent genes. In the present study, no differences were detected in SA or JA accumulation levels in the transgenic plants expressing CP when compared to the non-transgenic plants (Figure 2D). Therefore, these results suggest that CgCP suppresses the SA-sensing or downstream signaling pathway by altering one or more components involved in hormone cross-talk.

### CgCP expression stabilizes DELLA proteins and reduces growth of *Arabidopsis thaliana*

In recent years, the DELLA proteins have been shown to be involved in modulating cross-talk between hormonal and defense signaling pathways, including the balance of JA and SA signaling [29]. Here, we demonstrated that CgCP delayed the GA-mediated degradation of GFP-RGA (a DELLA protein) fusion protein (Figure 4B and C). Moreover, the expression of DELLA-target genes was increased in plants expressing CgCP. Recently; the impairment of E3 ligases has been suggested to be a strategy employed by viruses to protect proteins that are usually unstable [50]. Furthermore, geminiviral C2/L2 protein has been shown to interact with a component of COP9 signalosome (CSN complex) compromising the CSN activity over CUL1-based SCF ubiquitin E3 ligases [4]. Consequently, CUL-1 based ubiquitin ligases are altered in these plants. These ligases are involved in the regulation of many hormone signaling factors, among which are the DELLA proteins.

Based on these previous data, one possible speculation could be that CgCP alters some component of the proteasome machinery and, as a result, this would produce a delay in the GA-mediated degradation of DELLA proteins during CgCP expression. Alternatively, the effect on DELLA stabilization could be explained by the ability of TMV-Cg CP to alter a specific component of the GA-signaling pathway. For example, the P2 protein of RDV interacts with an ent-kaurene oxidase-like protein that is involved in gibberellin biosynthesis and, as a result, infected rice plants show a decrease of gibberellin GA1 levels compared to non-inoculated plants [3]. As a consequence of the reduced levels of GA, rice plants infected with RDV show reduced plant height compared to non-infected plants [3]. Importantly, these findings suggest that the change in GA levels or the alteration of GA-mediated signaling could explain some of the symptoms produced by plant viruses.

Here, we demonstrate that CgCP expression reduces plant growth both in seedlings and in adult plants when CgCP transcript levels are accumulated to high levels at early stages of plant development (Figure 3A, 3B, 3D). Taken together, in the present work, we demonstrated that CgCP stabilizes the DELLA proteins and therefore Arabidopsis exhibits a delay in flowering time as well as a reduction in plant growth.

### DELLA proteins negatively modulate antiviral defense signaling pathways

The role of DELLAs in the modulation of defense response to biotrophic bacterial pathogens has been previously characterized by Navarro et al. [29]. However, their role in viral signaling defense has not yet been studied**.** This specific area of study is particularly relevant because the SA-signaling components that are involved in antiviral resistance differ from those triggered by other pathogens. For example, the PR proteins are involved in the resistance to bacteria or fungi but do not seem to play a role in antivirus resistance [42]. In addition, the inhibition of viral accumulation by SA seems to be dependent on the action of specific host components. One of these proteins, *RDR1,* is a component of RNA silencing pathway. *RDR1* is needed for the maintenance of basal resistance to several RNA viruses, including TMV [51]. Moreover, the infection of *rdr1* mutant plants by TMV-Cg results in higher viral accumulation both in inoculated and in systemically infected leaves [34]. Besides the direct role of RDR1 on viral resistance, this protein may also play an indirect role regulating the expression of other defense-related genes in *N. tabacum*[52]. Particularly, the expression level of *AOX1A* is increased in WT plants after PVY infection, while the expression level of *AOX1A* is not increased in *rdr1* mutants after PVY infection. *AOX1A* has also been proposed to be involved in SA-induced viral resistance in susceptible tobacco plants [21].

In this work, we studied the expression of both *RDR1* and *AOX1A* in a quadruple-DELLA mutant and in *gai-1* mutant plants. Reduced levels of expression of *AOX1A* and *RDR1* genes were observed in the *gai-1* mutant plants when compared to the non-mutant Ler plants (Figure 5A). By contrast, the quadruple-*DELLA* mutants showed higher expression of *AOX1A* and *RDR1* than the non-mutant Ler plants (Figure 5A). Several reports have shown that reactive oxygen species (ROS) are involved in signaling defense events and potentiate SA signaling [53],[54]. Particularly, recent studies have shown that SA-induced *RDR1* expression is dependent on NPR1 protein and is enhanced by hydrogen peroxide [55],[56]. Furthermore, both SA and hydrogen peroxide are also involved in *AOX1A* induction [57],[58]. DELLA proteins increases the expression of genes encoding ROS- detoxification enzymes and consequently ROS levels are reduced [36]. Based on these findings, DELLA proteins may be attenuating *RDR1* and *AOX1A* expression by means of changing ROS levels. Moreover, these findings suggest that the stabilization of DELLA proteins during viral infection may be enhancing plants susceptibility to viruses.

### TMV-Cg virus stabilizes DELLA proteins and attenuates the induction of defense signaling during TMV-Cg infection

The DELLA proteins are stabilized during several abiotic and biotic stresses, [29],[59] and during viral protein expression [4]. However, the role of DELLA proteins has not been previously described during virus infection. Here, we observed that TMV-Cg virus delayed the GA-mediated DELLA degradation in *N. benthamiana* plants (Figure 6A and B). Moreover, we showed that TMV-Cg produced a delay in floral transition and reduced plant height in *Arabidopsis thaliana* (Figure 6C). This result is in agreement with the involvement of DELLA proteins in the growth inhibition response during stress.

**Figure 9:**
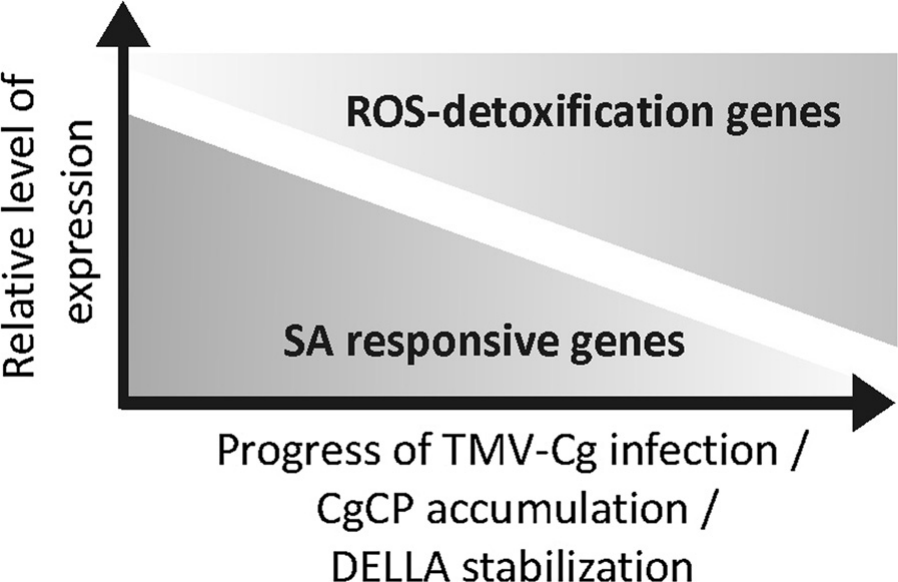
**Model proposed to explain the role of DELLA proteins in the modulation of SA-responsive genes during TMV-Cg infection.** As the virus infection progresses, CgCP accumulation stabilizes DELLA proteins and SOD-detoxification genes are upregulated. The stabilization of DELLA protein negatively modulates the expression of SA- responsive genes.

TMV-Cg stabilized DELLA proteins and in turn these proteins negatively modulated the expression of antiviral defense genes. These results led us to hypothesize that DELLA proteins could alter the gene expression profile during TMV-Cg infection. As expected, the expression of the same SA-responsive genes is reduced as TMV-Cg expression progresses (Figure 7A), while an increase of the expression of two DELLA-target transcript, *CSD1* and *CSD2*, is observed (Figure 7A). The expression profile of these genes remained unaltered at 9 dpi in quadruple-DELLA mutants infected with TMV-Cg compared to the non-infected quadruple-DELLA mutants (Figure 7B); which indicates a clear role of DELLA in tested genes alteration. Based on these results, we proposed a model to describe the role of DELLA proteins in the modulation of SA-responsive genes during TMV-Cg infection (Figure 9). As the virus infection progresses, CgCP stabilizes DELLA proteins. This stabilization enhances the expression of SOD-detoxification genes and negatively modulates the expression of SA- responsive genes.

Moreover, based on the results presented here, it can be suggested that CgCP produces an increasing enhancement of host susceptibility by attenuating the expression of DELLA-regulated SA-responsive defense genes. In agreement with this hypothesis, we observed that infected quadruple-DELLA mutants (in which defense genes where overexpressed) accumulated approximately 100-fold less virus CP mRNA than infected WT-Ler plants. However, the level of viral accumulation remained unaltered in infected *gai-1* mutants in comparison with infected WT-Ler plants (Figure 8). Similarly, previous observations of other groups have shown that *NahG* plants (SA deficient plants) inoculated with a Tobamovirus (ORMV) show no enhanced susceptibility to virus in comparison to WT plants [24]. Huang et al. suggested that viruses could counteract SA-defense responses during compatible interactions [24]. This counteraction therefore could explain why the depletion of SA does not enhance virus susceptibility.

## Conclusions

In summary, the modulation of hormone signaling pathway by plant viral proteins seems to be another mechanism to counteract host defenses. In particular, this work demonstrates that CgCP attenuates the signaling pathways downstream SA by stabilizing DELLA proteins. Moreover, this research shows that the DELLA proteins are stabilized during virus infections, thus modulating the level of SA antiviral genes. An implication of this is the possibility that the DELLA proteins may enhance the plant susceptibility to TMV-Cg virus infection. Further studies on the role of DELLA proteins during virus infections will add to the understanding of the modulation of SA signaling by Tobamovirus.

## Methods

### Plant materials

The *Arabidopsis thaliana* lines used in this study were derived either from the Landsberg erecta (Ler) ecotype (pRGA::GFP-RGA, *gai-1* (TAIR stock number: CS63) and quadruple-DELLA mutant line) [60],[40] or Columbia (Col-0) ecotype (CP#72 transgenic line) [30]. The pRGA::GFP-RGA x CP#72 lines were generated by crossing the corresponding lines. Self crosses were performed to obtain homozygous double transgenic plants, confirmed by a genomic polymerase chain reaction (PCR)-based screening.

### Growth experiments of mature plants

*Arabidopsis thaliana* seeds were sown onto pots and stratified at 4°C for 3 days to coordinate germination prior to the transference to the AR-95 L Controlled Environmental Chamber (Percival, Perry, IA). Plants were grown under standard conditions. The relative humidity was maintained at 60 to 70%. Plants were watered by subirrigation as needed, usually every two to three days, depending on growth stage. The day length was set to be 16 h per day. Daytime and nightime temperatures were maintained at 23 and 21°C, respectively. The average light intensity at the top of the pots was 100 μmol m^−2^ sec^−1^. To analyze the effect of TMV-Cg CP over growth, the CP#72 plants grown to stage 1.08 were treated either with 61.3 μM MOF or water. Subsequently, the plant height was measured after six weeks.

### Growth experiments of seedlings

All seeds were surface sterilized and placed on Murashige Skoog (MS) (half-concentrated MS medium) plates at 4°C for three days to synchronize germination. The plates were then placed in a growth chamber (20°C; 16:8 h light/dark photoperiod). Upon completion of germination, the seedlings were treated with 61.3 μM MOF or water. The fresh weight of MOF-treated seedlings and mock-treated seedlings was measured 10 days afterwards.

### CgCP induction and SA treatment

Mature *Arabidopsis thaliana* plants (1.08 stage) grown in soil were treated (drenched) either with 61.3 μM MOF or water 48 h prior to SA treatments. Then, the plants were sprayed with 0.5 ml of either 0.5 mM salicylic acid (SA) or water. Whole rosettes were sampled 24 h after SA treatment, immediately frozen in liquid nitrogen and stored at −80°C until RNA isolation.

### Detection of GFP–RGA by fluorescence microscopy

The levels of GFP-RGA were determined by fluorescence confocal microscopy, as previously described [40]. Five-day old *pRGA:GFP-RGA x CP#72* seedlings were grown on half MS medium, treated with 61.3 μM MOF for three days, and subsequently treated for 1 h with 10 μM GA3.

### SA and JA Measurements

The determination of SA and JA contents was performed as previously described [19]. *Arabidopsis thaliana* Col-0 and CP#72 leaves were treated with 61.3 μM MOF at growth stage 1.08. Then, samples were collected at 0, 24, 48 and 72 h after MOF treatment.

### *Nicotiana benthamiana* transient expression assays

The *Nicotiana benthamiana* plants were inoculated with TMV-Cg virus. The agroinfiltration was performed six days post infection (dpi), as previously described [61]. The GA treatment and YFP-GAI visualization were performed as described by Lozano-Durán [4].

### Quantitative analysis of GFP and YFP accumulation

Twenty random nuclei from five different plants for each treatment were analyzed for the quantification of GFP/ YFP levels (intensity of color) accumulated in Arabidopsis and N. benthamiana cells using the ImageJ Software (http://imagej.nih.gov/ij/). All visible nuclei were quantified, in cases in which the visible nuclei were fewer than twenty. All images were captured using the same microscope settings and processed in the same manner. Statistical comparisons were performed by one-way ANOVA with Tukey post-test using Infostat statistical software (InfoStat version 2008. Grupo InfoStat. FCA, Universidad Nacional de Cordoba). The significance level for both post-tests was α = 0.05.

### Virus infection assays

The third expanded leaf of each plant (1.08 stage) was dusted with carborundum. Subsequently, 5 μl of semi-purified TMV-Cg virus diluted in 20 mM phosphate buffer (pH 7) were added and the surface of the leaf was gently abraded. The mock-inoculated plants were buffer-rubbed. Samples of systemic leaves were taken at 5, 7 and 9 dpi. The leaves were frozen in individual tubes in liquid nitrogen and stored at −80°C until RNA extraction.

### Quantitative real-time polymerase chain reaction (RT-qPCR)

Total RNA was isolated from frozen *Arabidopsis thaliana* leaf tissues using Trizol Reagent (Invitrogen) and subsequently treated with DNAse I (Invitrogen). For messenger-RNA detection, the first-strand cDNA was synthesized using MMLV (Invitrogen) according to manufacturer’s instructions. All RT-qPCR experiments were carried out in an ABI Prism 7500 Real Time PCR System (Applied Biosystems) equipment, the experimental conditions used followed MIQE (Minimun information for publication of quantitative real time PCR experiments) requirements (see Table 1 for more details). *Ubiquitin5 (UBQ5*, NM-116090) was used as an internal reference gene. The oligonucleotide primer set used for RT- qPCR are listed in Table 2. RT-qPCR data analysis and primer efficiencies were obtained using LinReg PCR software [62]. A reference gene was used to standardize the expression of a given target gene; then, a ratio between treatments was calculated using the algorithm developed by Pfaffl et al. [63]. Relative expression ratios and statistical analysis were performed using fgStatistics software interface (J. A. Di Rienzo, personal communications).The cut-off for statistically significant differences was set as *P* value < 0.05, indicated as *.

**Table 1:** Experimental conditions used in RT-qPCR based on MIQE requirements

**Experimental design**	
Control groups	CP#72 water treated plants/mock infected plants
Treatment groups	CP#72 methoxyfenozide treated plants/TMV-Cg infected plants
**Sample**	
Type of sample	Arabidopsis leaves
Procesing procedure	Nitrogen liquid homogenization
Sample frozen conditions	−80°C
**RNA extraction**	
Procedure	Acid Phenol extraction
Reagents	TRIzol
Details of Dnasa treatment	DNAsa I Amp Grade, 15 min at room temperature
Contamination assesment	<3%
Nucleic acid quantification	
Instrument and method	NanoDrop instrument
Purity (A260/A280)	>1.8
RNA integrity	Analysed by agarose gel electrophoresis
**Reverse transcription**	
Complete reaction conditions	Reaction was performed as recomendad by Invitrogen®
Amount of RNA and reaction volume	1 μg of RNA, 20 μl
Priming oligonucleotide	Oligo d(T) 20 primers (Invitrogen®) and random primers
Reverse transcriptase	MMLV® III Reverse Transcriptase
Temp and time	1 hour, 50°C
**qPCR protocol**	
Complete reaction conditions	5 min 95°C , (30 seg 95°C, 1 min 60°C) x 40 cycles
Reaction volume and amount of cDNA	20 μl reaction, 20–200 ng de RNA
Primer, Mg and dNTPs concentration	3 mM Mg2+, 200nM primers, 0,2 mM de dNTPs
Polymerase	Taq platinum, Invitrogen
Buffer	20 mM Tris–HCl (ph 8.4), 50 mM Kcl
Manufacturer of qPCR instrument	ABI 7500, Applied Biosystems
**qPCR validation**	
Specificity	Analysed by agarose gel and melting curve parameters on each qPCR run
Method of PCR efficiency calculation	Mean PCR efficiency per amplicon calculated by LingRegPCR program [62].
**Data analysis**	
qPCR analysis program	LinRegPCR program
Method of Cq determination	LinRegPCR program
Outlier identification	LinRegPCR program
Justification of number and choice of reference genes	3 references genes tested (*EF1*α, *UBQ5*, *GAPC1*) for stability using three stability algorithms. *UBQ5* was selected as the reference gene.
Description of normalization methods	Pfaffl’s mathematical model [63].
Number of technical replicates	3
Statistical method	Permutation test
Software	Fg Satistics
Repeatability (intraassay variation) C_q_ SD error	0.8

**Table 2:** List of used primers

**Locus/Accesion number**	**Gene name**	**Primers sequences**
At3g22370	*AOX1A*	CCGACGATTGGAGGTATGAGATT
		CCGGTGGATTCGTTCTCTGTTT
At3g45640	*MPK3*	TACACGGATTTTCCGGCGGTGGA
		TCGGAGGACGATACTTAGA
At5g26920	*CBP60g*	CGCACCAAGCCGTTGAAACGATG
		CTGGCTCTTGGAGATCCCA
At2g38470	*WRKY33*	GTTTCAGTCCCTCTCTTTTT
		GTTCTTGTCTCCTTCATTTA
At2g30250	*WRKY25*	ATGTCTTCCACTTCTTTCACC
		GTACCCACCAAAACTCGAC
At3g56400	*WRKY70*	CACCAACGCAGAAACTCCCA
		TTCTCCGTGGACGAACCATG
At1g14790	*RDR1*	TGGAATTGGGATGAGGAAACTG
		TCGTCCTTGCGGAGAATGC
At1g08830	*CSD1*	CCAAAGAGAGACGAAGCA
		GCCTTTCTGATCTCAGGA
At2g28190	*CSD2*	GCTCCAGAAGATGAGTGCCGTCA
		CCACCCTTTCCGAGGTCATCCTT
NM-116090	*UBQ5*	CGGACCAGCAGCGATTGATT
		ACGGAGGACGAGATGAAGCG
At3g63010	*GID1b*	CAGCTTCACTCATTCCTCCGCCA
		GCGTTCCATCCATCGTCGTAAGC
At4g36410	*UBC17*	GGCAAACCAATCCTCCTTCT
		TTGGCGTAAAGAGTTCCTGG
At5g50915	*bHLH137*	TCGAGCTAGAAGAGGCCAAGC
		TCACAGCCCGGAACAAGGT
At4g23060	*IQD22*	TTCCTCATACCCATCGCTACCG
		TCCTCATCCTCCACATCG
D38444	TMV-Cg replicase	GAAGTTCACTTAGAAGAGTTGC
		AACGGCCTCATCTAACTG
D38444	TMV-Cg CP	TGTCGCAATCGTATCAAAC
		CTGTATCTGGAAACCGCTG

### Statistic analysis

The following variables were analyzed between plant groups: SA and JA levels, plant height, flowering time and fresh weight. All statistical comparisons were performed by one-way ANOVA with Tukey post-test using Infostat statistical software (InfoStat version 2008. Grupo InfoStat. FCA, Universidad Nacional de Cordoba). The significance level for all post-tests was α = 0.05.

## Abbreviations

CaMV: Cauliflower mosaic virus

CgCP: TMV-Cg coat protein

CP-MR: Coat protein-mediated resistance

GA: Gibberellic acid

JA: Jasmonic acid

MIQUE: Minimum Information for Publication of Quantitative Real-Time PCR Experiments

MOF: Methoxyfenozide

PR1: Pathogenesis-related

RDV: Rice dwarf virus

RDR1: RNA-dependent RNA polymerase 1

SA: Salicylic acid

TMV: Tobacco mosaic virus

WT: Wild type plants

## Competing interests

The authors declare that they have no competing interests.

## Authors’ contributions

Conceived and designed the experiments: MCR, SA. Performed the experiments: MCR GC DZ. Analyzed the data: MCR SA. Contributed reagents/materials/analysis tools: MCR GC DZ CAM. Wrote the paper: MCR SA. Revising the article: MCR GC DZ SA. Both authors read and approved the final manuscript.

## Additional files

## Supplementary Material

Additional file 1: **Expression of*WRKY70*, *RDR1*and*AOX1A*in non-transgenic MOF treated Col-0 plants compared to the expression levels in non- transgenic water treated Col-0 plants.** Description of the data: The samples were collected at 48 h after MOF treatment. The expression level in non- transgenic water treated Col-0 plants was arbitrarily set to one (Log_2_(1) = 0). Asterisks indicate statistically significant differences (* = P values < 0.05). Click here for file

Additional file 2: **CgCP expression reduces plant growth and delays the timing of floral transition in CP#71 transgenic line.** Description of the data: Representative WT Col-0 and CP#71 transgenic 6-week-old plants. Measurements were taken in CP#71 and WT Col-0 plants. A) Bar graph showing plant height (6-weeks-old) and B) floral transition (number of days to first flower). Each column represents the mean of 25 plants ± SE. Click here for file

Additional file 3: Representative Col-0 seedlings of 10 days of age grown for seven days in presence of MOF (MOF) or water (−). Click here for file

Additional file 4: **Expression of DELLA targets genes in quadruple-DELLA and*gai-1*adult plants.** Relative mRNA levels of individual genes in quadruple-DELLA mutant plants and *gai-1* mutant plants were calculated in comparison to WT-Ler plants. Description of the data: Expression level in WT-Ler plants was arbitrarily set to one (Log_2_(1) = 0). The means of four replicates of quantitative RT-qPCR ± SE are shown. Asterisks indicate statistically significant differences (* = P values < 0.05). Click here for file

Additional file 5: **Representative CP#72/RGAp::GFP-RGA primary seedling roots observed under fluorescence microscope.** Description of the data:A) GFP fluorescence observed in water- treated seedlings; B) GFP fluorescence observed in water- treated seedlings subsequently treated with 10 μMGA_3_; C) GFP fluorescence observed in MOF treated seedlings; D) GFP fluorescence observed in MOF-treated seedlings subsequently treated with 10 μMGA_3_. Click here for file

Additional file 6: **Expression analysis of a subset of defense genes using Genevestigator.** Description of the data: mRNA expression level of WT plants treated with flg22 (WT + flg22) was set to one. Relative mRNA levels of individual genes in *gai-1* mutant plants treated with flg22 ( *gai-1* + flg22) were compared to WT plants treated with flg22. Color scale corresponds to the degree to which the expression was below the control (WT + flg22). Click here for file

Additional file 7: **Representative*N. benthamiana*leaves observed under fluorescence microscope.** Description of the data: A) YFP fluorescence observed in non-infected N benthamiana leaves; B) YFP fluorescence observed in non-infected N. benthamiana subsequently treated with 100 μ MGA_3_; C) YFP fluorescence observed in infected N benthamiana leaves; D) YFP fluorescence observed in infected N benthamiana leaves treated with 100 μMGA_3_. Click here for file

Additional file 8: **DELLA proteins reduce the level of TMV-Cg replicase.** Description of the data: Expression level in WT-Col-0 plants was arbitrarily set to one (Log_2_(1) = 0). The means of four replicates of quantitative RT-qPCR ± SE are shown. Asterisks indicate statistically significant differences (* = P values < 0.05). Click here for file
