# Supplementary figures and images for: TMV-Cg Coat Protein stabilizes DELLA proteins and in turn negatively modulates salicylic acid-mediated defense pathway during Arabidopsis thaliana viral infection
Source: BMC Plant Biol. 2014 Aug 3;14:210. doi: 10.1186/s12870-014-0210-x (PMC4422269; doi:10.1186/s12870-014-0210-x)

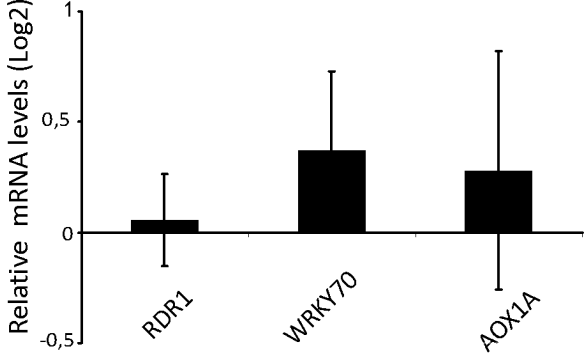

Supplement: Additional file 1: — Expression ofWRKY70, RDR1andAOX1Ain non-transgenic MOF treated Col-0 plants compared to the expression levels in non- transgenic water treated Col-0 plants. Description of the data: The samples were collected at 48 h after MOF treatment. The expression level in non- transgenic water treated Col-0 plants was arbitrarily set to one (Log2(1) = 0). Asterisks indicate statistically significant differences (* = P values < 0.05). [file s12870-014-0210-x-S1.pdf]

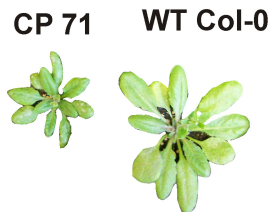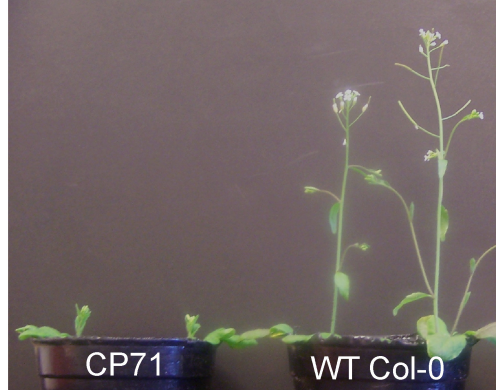

**A**

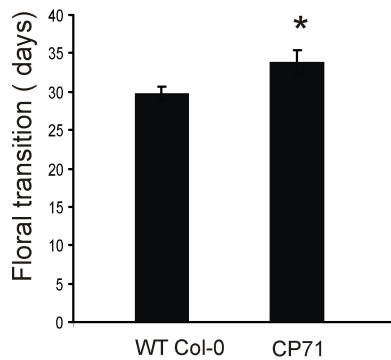

**B**

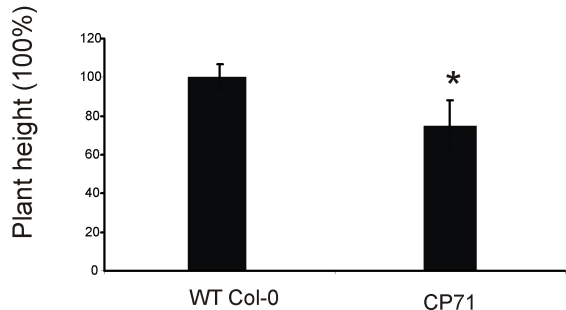

Supplement: Additional file 2: — CgCP expression reduces plant growth and delays the timing of floral transition in CP#71 transgenic line. Description of the data: Representative WT Col-0 and CP#71 transgenic 6-week-old plants. Measurements were taken in CP#71 and WT Col-0 plants. A) Bar graph showing plant height (6-weeks-old) and B) floral transition (number of days to first flower). Each column represents the mean of 25 plants ± SE. [file s12870-014-0210-x-S2.pdf]

1cm

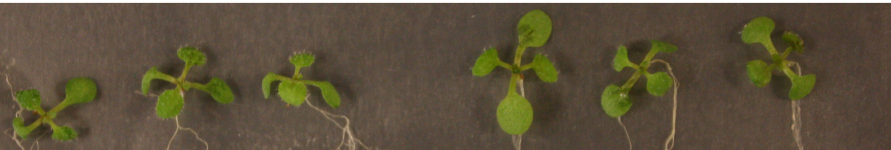

Col-0+ water

Col-0+ MOF

Supplement: Additional file 3: — Representative Col-0 seedlings of 10 days of age grown for seven days in presence of MOF (MOF) or water (−). [file s12870-014-0210-x-S3.pdf]

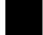

quadruple-DELLA

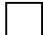

*gai-1*

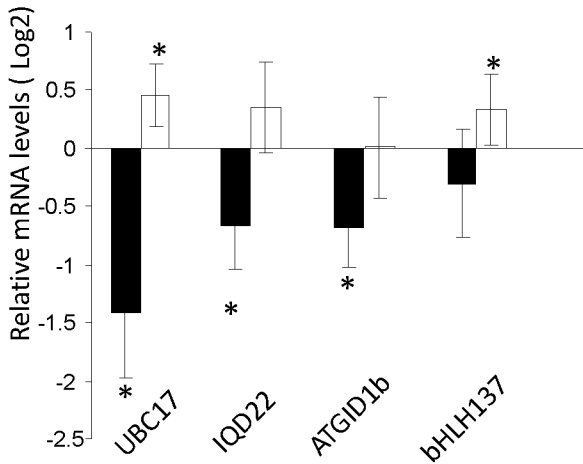

Supplement: Additional file 4: — Expression of DELLA targets genes in quadruple-DELLA andgai-1adult plants. Relative mRNA levels of individual genes in quadruple-DELLA mutant plants and gai-1 mutant plants were calculated in comparison to WT-Ler plants. Description of the data: Expression level in WT-Ler plants was arbitrarily set to one (Log2(1) = 0). The means of four replicates of quantitative RT-qPCR ± SE are shown. Asterisks indicate statistically significant differences (* = P values < 0.05). [file s12870-014-0210-x-S4.pdf]

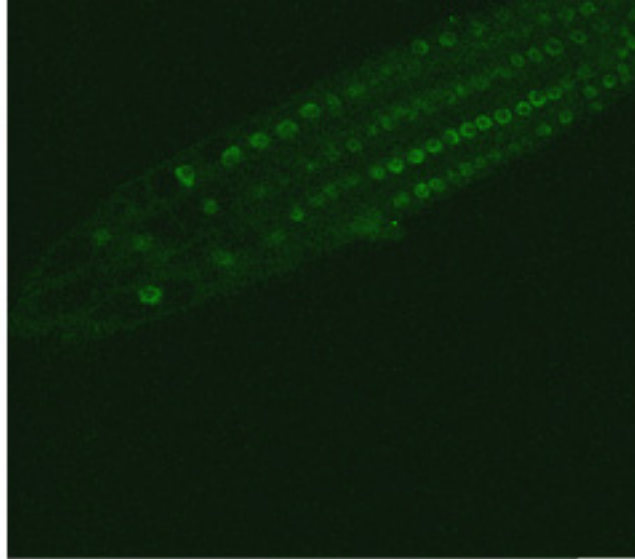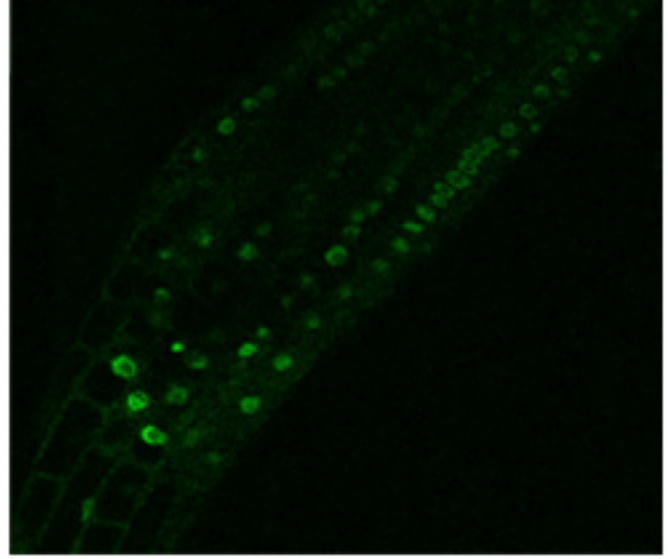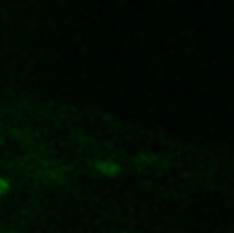

**B**

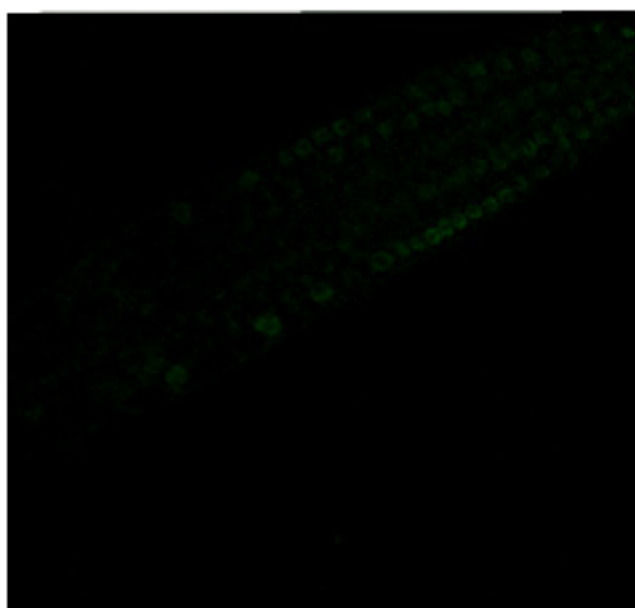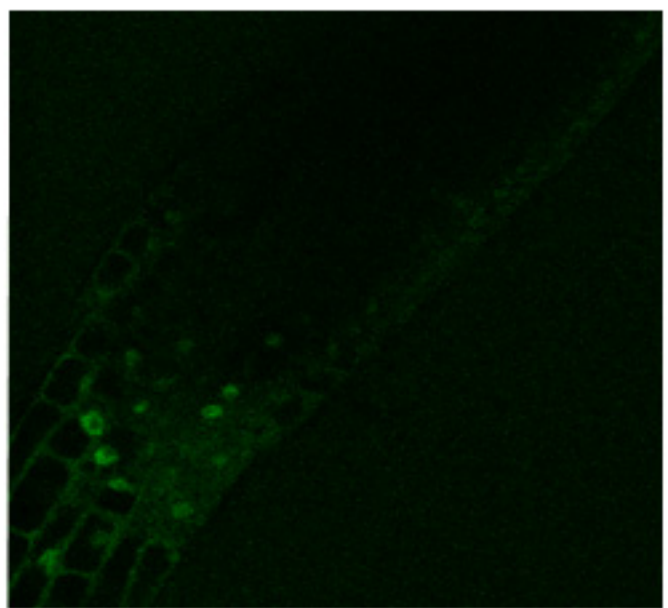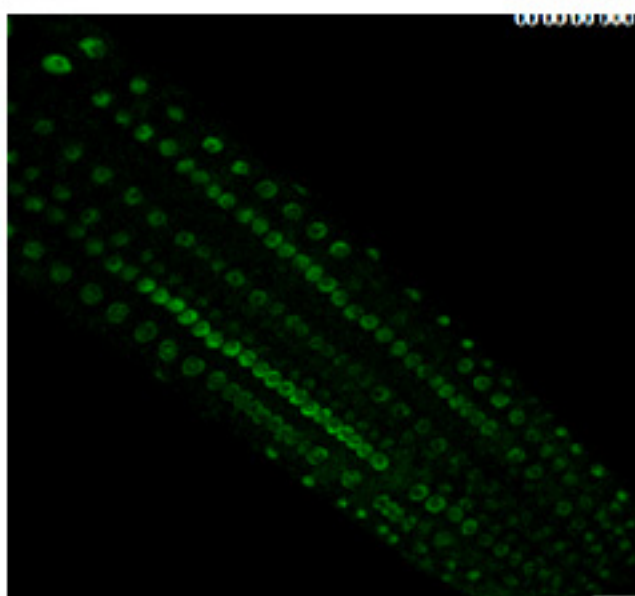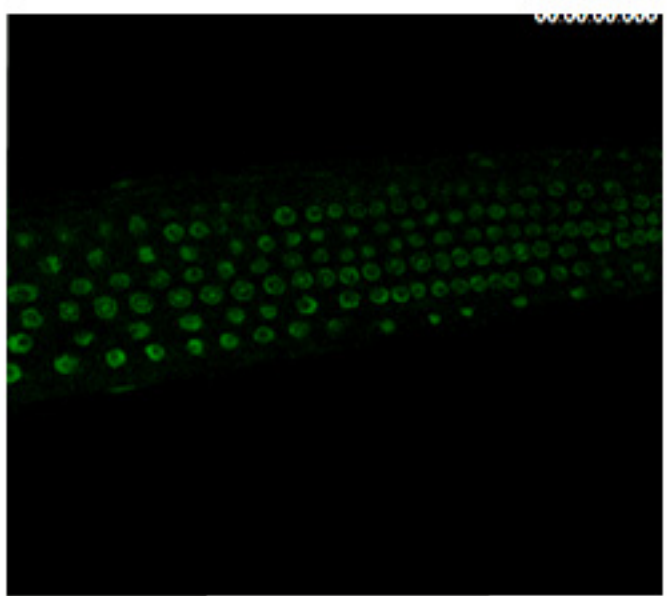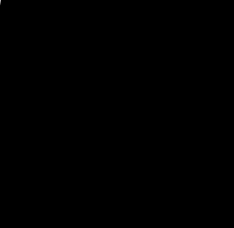

**D**

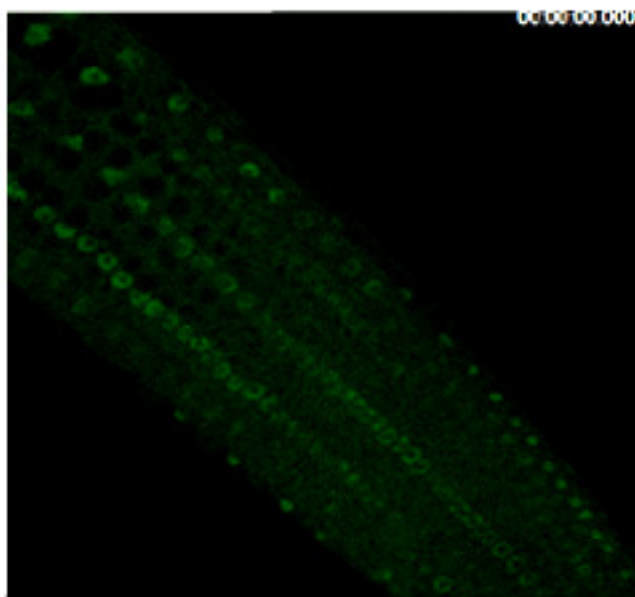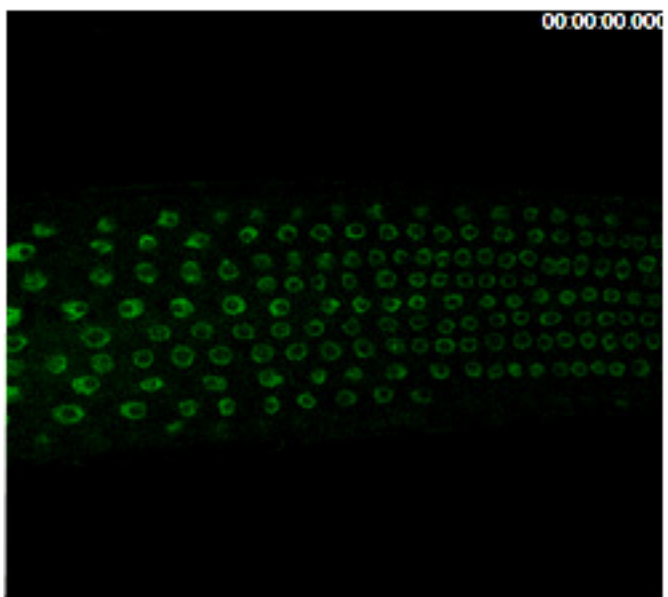

Supplement: Additional file 5: — Representative CP#72/RGAp::GFP-RGA primary seedling roots observed under fluorescence microscope. Description of the data:A) GFP fluorescence observed in water- treated seedlings; B) GFP fluorescence observed in water- treated seedlings subsequently treated with 10 μMGA3; C) GFP fluorescence observed in MOF treated seedlings; D) GFP fluorescence observed in MOF-treated seedlings subsequently treated with 10 μMGA3. [file s12870-014-0210-x-S5.pdf]

WT+flg22

*gai-1* +flg22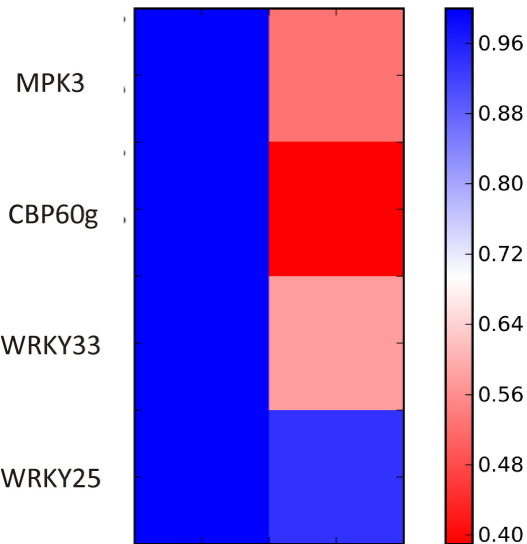

Supplement: Additional file 6: — Expression analysis of a subset of defense genes using Genevestigator. Description of the data: mRNA expression level of WT plants treated with flg22 (WT + flg22) was set to one. Relative mRNA levels of individual genes in gai-1 mutant plants treated with flg22 ( gai-1 + flg22) were compared to WT plants treated with flg22. Color scale corresponds to the degree to which the expression was below the control (WT + flg22). [file s12870-014-0210-x-S6.pdf]

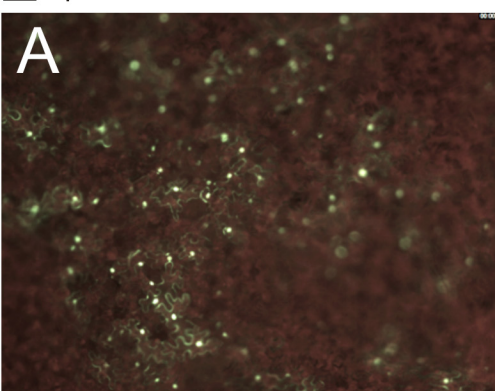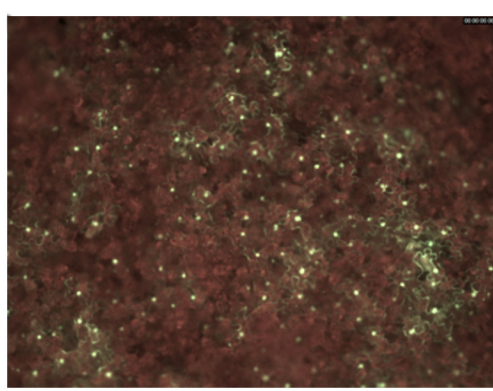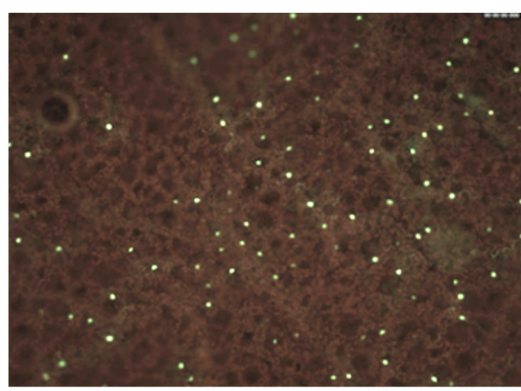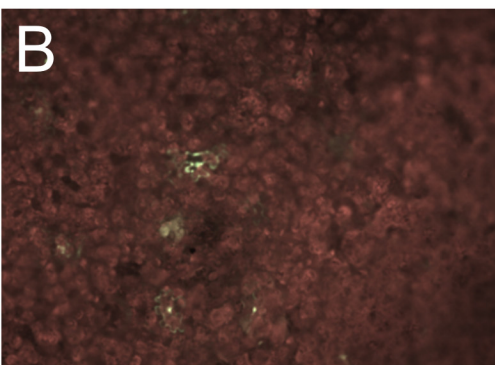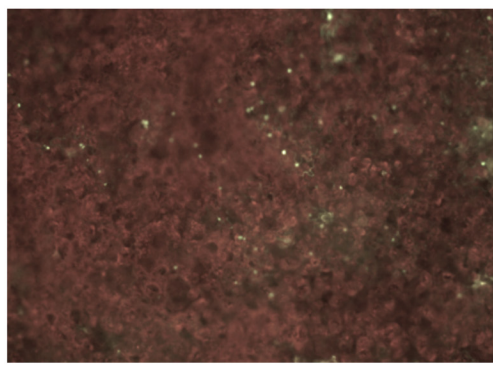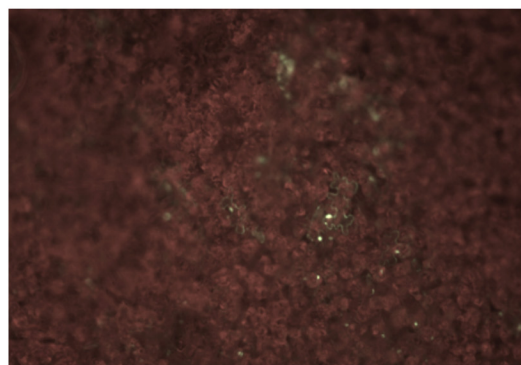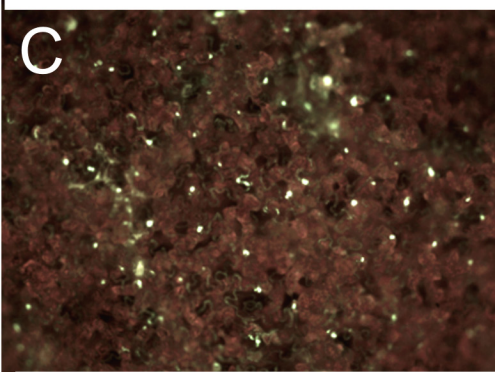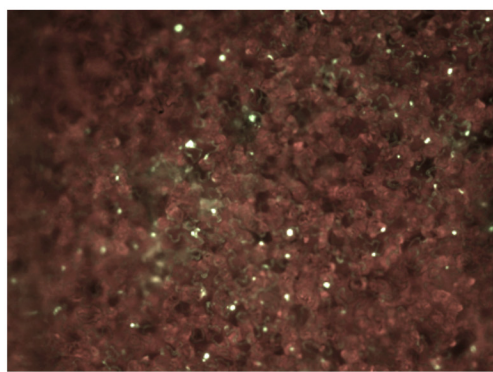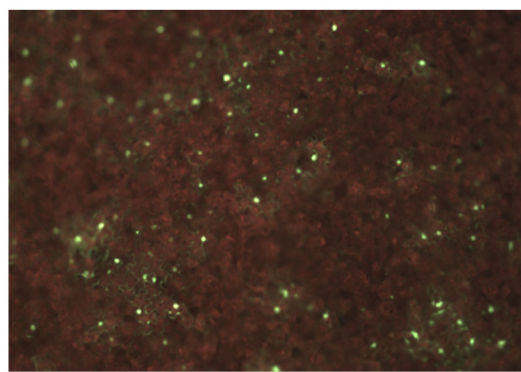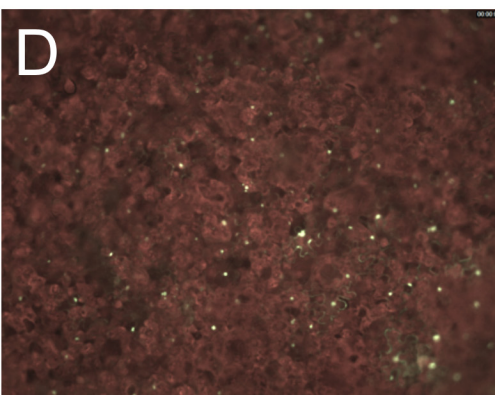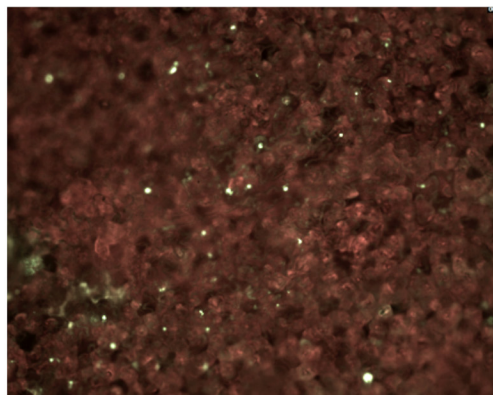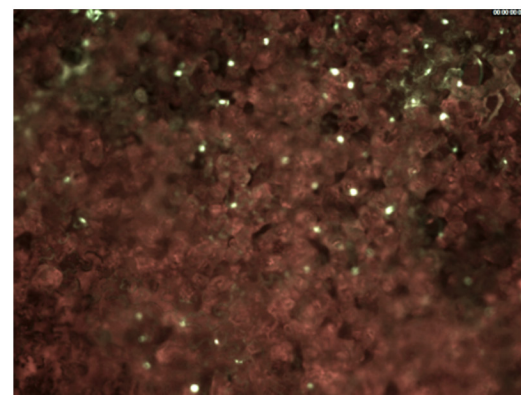

Supplement: Additional file 7: — RepresentativeN. benthamianaleaves observed under fluorescence microscope. Description of the data: A) YFP fluorescence observed in non-infected N benthamiana leaves; B) YFP fluorescence observed in non-infected N. benthamiana subsequently treated with 100 μ MGA3; C) YFP fluorescence observed in infected N benthamiana leaves; D) YFP fluorescence observed in infected N benthamiana leaves treated with 100 μMGA3. [file s12870-014-0210-x-S7.pdf]

Relative mRNA levels (Log2)

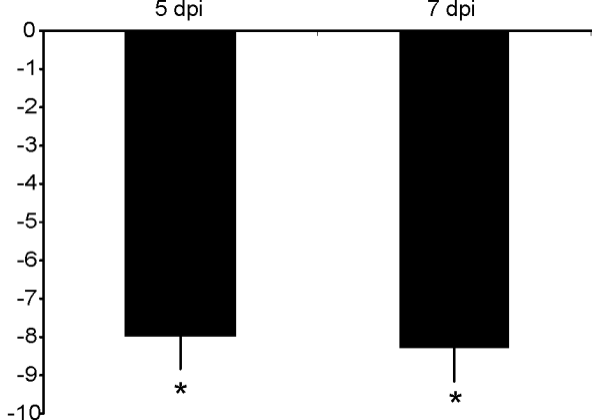

Supplement: Additional file 8: — DELLA proteins reduce the level of TMV-Cg replicase. Description of the data: Expression level in WT-Col-0 plants was arbitrarily set to one (Log2(1) = 0). The means of four replicates of quantitative RT-qPCR ± SE are shown. Asterisks indicate statistically significant differences (* = P values < 0.05). [file s12870-014-0210-x-S8.pdf]
